# Vascular function of the T3/modern clade *WUSCHEL-Related HOMEOBOX* transcription factor genes predate apical meristem-maintenance function

**DOI:** 10.1186/s12870-022-03590-0

**Published:** 2022-04-25

**Authors:** Christopher E. Youngstrom, Kelley A. Withers, Erin E. Irish, Chi-Lien Cheng

**Affiliations:** Department of Biology, 129 E. Jefferson St. Iowa City, Iowa, 52242-1324 USA

**Keywords:** WOX, CrWUL, Vascular cambium, Apical meristem, Fern, Ceratopteris, Arabidopsis, *Selaginella kraussiana*

## Abstract

**Background:**

Plants have the lifelong ability to generate new organs due to the persistent functioning of stem cells. In seed plants, groups of stem cells are housed in the shoot apical meristem (SAM), root apical meristem (RAM), and vascular cambium (VC). In ferns, a single shoot stem cell, the apical cell, is located in the SAM, whereas each root initiates from a single shoot-derived root initial. *WUSCHEL-RELATED HOMEOBOX* (*WOX*) family transcription factors play important roles to maintain stem-cell identity. *WOX* genes are grouped phylogenetically into three clades. The T3WOX/modern clade has expanded greatly in angiosperms, with members functioning in multiple meristems and complex developmental programs. The model fern *Ceratopteris richardii* has only one well-supported *T3WOX*/modern *WOX* gene, *CrWUL*. Its orthologs in *Arabidopsis*, *AtWUS*, *AtWOX5*, and *AtWOX4*, function in the SAM, RAM, and VC, respectively. Identifying the function of *CrWUL* will provide insights on the progenitor function and the diversification of the modern *WOX* genes in seed plants.

**Results:**

To investigate the role of CrWUL in the fern, we examined the expression and function of *CrWUL* and found it expresses during early root development and in vasculature but not in the SAM. Knockdown of CrWUL by RNAi produced plants with fewer roots and fewer phloem cells. When expressed in *Arabidopsis* cambium, *CrWUL* was able to complement *AtWOX4* function in an *atwox4* mutant, suggesting that the WOX function in VC is conserved between ferns and angiosperms. Additionally, the proposed progenitor of *T3WOX* genes from *Selaginella kraussiana* is expressed in the vasculature but not in the shoot apical meristem. In contrast to the sporophyte, the expression of *CrWUL* in the gametophyte exhibits a more general expression pattern and when knocked down, offered little discernable phenotypes.

**Conclusions:**

The results presented here support the occurrence of co-option of the *T3WOX/*modern clade gene from the gametophyte to function in vasculature and root development in the sporophyte. The function in vasculature is likely to have existed in the progenitor of lycophyte *T3WOX*/modern clade genes and this function predates its SAM function found in many seed plants.

**Supplementary Information:**

The online version contains supplementary material available at 10.1186/s12870-022-03590-0.

## Background

Meristems of plant bodies house self-renewing stem cells. In seed plants, these centers include the shoot apical meristem (SAM), root apical meristem (RAM), and the vascular cambium (VC). Stem cells within these tissues divide to produce two daughter cells; and once displaced outside the niche actively proliferate to form new organs or differentiated tissues [1–4]. To achieve a balanced population of stem cells and differentiating cells, hormonal and other cellular signals regulate specific families of transcription factors, such as *WUSCHEL-Related HOMEOBOX* (*WOX*) transcription factors, *SHOOT-MERISTMELESS* (*STM*), and *SHORTROOT* (*SHR*) in the apical meristems of *Arabidopsis thaliana* [5–7].

In the SAM, RAM, and VC of *Arabidopsis*, *WOX* genes maintain the size of the stem cell pool in response to auxin, cytokinin and/or CLAVATA signaling [8]. In the SAM, the AtWUS protein is expressed in the organizing center (OC) where it acts non-cell autonomously in the central zone (CZ), a few cell layers above, to maintain the stem cell fate [9]. This SAM maintenance model is likely conserved in the monocot maize [10]. The stem cells of the RAM are also maintained non-cell autonomously by AtWOX5, which moves from the quiescent center (QC) outward to surrounding stem cells [11, 12]. Recent results show that although mobile, movement is not required for AtWOX5 to inhibit stem cells from differentiation [13]. While AtWUS and AtWOX5 are mobile, AtWOX4 maintains VC stem cells in a cell-autonomous fashion [14, 15].

All land plants examined contain *WOX* genes [16, 17]. In a recently updated phylogeny of WOX proteins in Viridiplantae [17], three ancient superfamilies emerged, the Type 1 (T1WOX), Type 2 (T2WOX), and Type 3 (T3WOX) clades, which represent the previously named ancient, intermediate, and modern clades, respectively [16] with minor differences. These include the assignment of a lycophyte *WOX* gene as sister to the T2WOX and T3WOX clades and the absence of lycophyte and fern sequences from the T2WOX clade. In addition, four lycophyte *WOX* genes, three from *Isoetes* and one from *Selaginella moellendorfii*, *SmWOXII*, are now sister to the T3WOX clade. The *SmWOXII*’s ortholog in *Selaginella kraussiana*, *SkWOX11C* (Additional file 1: Fig. S1), has been shown to express in various sporophyte tissues [18]. By this new convention, each clade has members from as early as lycophytes and could allow for a clearer trajectory of *WOX* gene evolution. Despite the differences, both phylogenetic trees place three fern genes, one from *Ceratopteris richardii* (*CrWUL*) and two from *Azolla filiculoides* (Azfi_s0343.g065738, Azfi_s0051.g031311), firmly within the T3WOX/modern clade. As the sister clade to seed plants, ferns offer a unique opportunity to investigate whether the fern *T3WOX*/modern gene plays a function in the fern SAM and subsequently uncover the ancestral function of the T3WOX/modern clade progenitor.

Unlike *Arabidopsis* and other seed plants, most extant ferns have a single stem cell at the apex of the SAM called the shoot apical cell. As in *Arabidopsis*, the fern sporophyte SAM contains two regions that comprise distinct transcriptional profiles [19], a core region and a shoot apical cell (reviewed by [20]); however, the domains within the core region are not defined. How the shoot apical cell maintains its identity and what the roles, if any, the core region plays in the shoot apical cell maintenance are unknown. What role could a *T3WOX*/modern gene play in stem cell maintenance of a fern, despite the contrast in meristem organization?

In the fern model *Ceratopteris,* only one *T3WOX*/modern gene is well supported, *CrWUL*, has been found. It would be reasonable to speculate that *CrWUL* functions in the meristem or even maintains apical cell identity. The CrWUL protein has been shown to move and exhibit stem-cell maintenance functions in *Arabidopsis* SAM and RAM only after it is truncated and expressed under the control of *AtWUS* or *AtWOX5* promoter, respectively [21]. Those authors proposed that these two *Arabidopsis T3WOX*/modern genes have evolved through a two-step selection to acquire the stem-cell maintenance function in the SAM and RAM; the first was stem-cell maintenance activity and the second was intercellular mobility. However, in *Ceratopteris*, previous studies showed that *CrWUL* is expressed in the leaf vasculature [22], the merophytes of lateral roots [16] and adventitious roots [23] but not in the root apical cell [16]. These results suggest that *T3WOX/*modern genes were recruited to function in vascular tissues before the divergence of ferns and seed plants from the land plant phylogeny. Here, we show that *CrWUL* expresses and functions in the vascular tissue but not in the SAM of *Ceratopteris*. In addition, we provide evidence that the progenitor of *T3WOX*/modern genes were present in the last common ancestor of lycophyte and fern*.* Finally, we show that CrWUL can replace AtWOX4 function in *Arabidopsis* cambium cells. These results support the hypothesis that *T3WOX/*modern genes acquired apical stem-cell maintenance activity by first being recruited to function in vascular tissues.

## Results

### Expression of *CrWUL* during gametophyte and sporophyte development

**Fig. 1:**
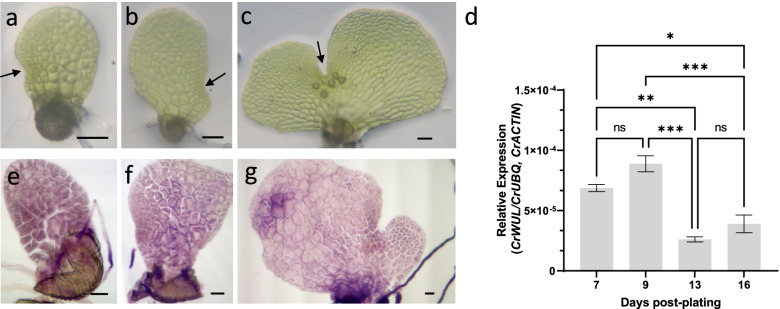
Expression of *CrWUL* in the gametophyte generation of *C. richardii*. (**a-c**) Representative wild-type hermaphrodite gametophytes at 7-, 9-, and 13-days post plating. (**d**) Expression of *CrWUL* during gametophyte development measured by RT-qPCR (mean ± SEM; *, *P* ≤ 0.05; **, *P* ≤ 0.01; ***, *P* ≤ 0.001; *N* = 3). (**e–g**) Whole mount in situ hybridization of 7-day (**g**), 9-day (**h**), and 13-day (**i**) old hermaphroditic gametophytes. Black arrowhead in (a-c) indicates notch meristem. Scale Bars = 0.05 mm

Because ferns have meristems not only in the sporophyte generation, but also in the gametophyte generation, the expression of *CrWUL* was examined in both generations. Initially, we determined the expression levels of *CrWUL* in gametophytes at different developmental stages. Hermaphroditic gametophytes establish the notch meristem 7-days post plating (dpp) and reach sexual maturity by 13-dpp (Fig. 1a-c). Expression of *CrWUL* increased slightly during sexual maturation in 7- to 9-dpp gametophytes and decreased by more than half after sexual maturation in 13-dpp gametophytes and remains low from 13- to 16-dpp (Fig. 1d). Whole mount in situ hybridization was performed to localize *CrWUL* transcripts during gametophyte development (Fig. 1e-g). At 7- and 9-dpp, *CrWUL* expression is present throughout the hermaphroditic gametophyte (Fig. 1e, f). After the establishment of the notch meristem, expression decreases in the hermaphrodite thallus (Fig. 1g) and becomes difficult to discern from background (see sense probe images, Additional File 1: Fig. S2), suggesting a drastic decline of *CrWUL* levels. In the male gametophyte, *CrWUL* expression was not detected by whole-mount in situ (Additional File 1: Fig. S2). Thus, expression of *CrWUL* was observed in the hermaphroditic gametophyte prior to sexual maturation and declined subsequently.

**Fig. 2:**
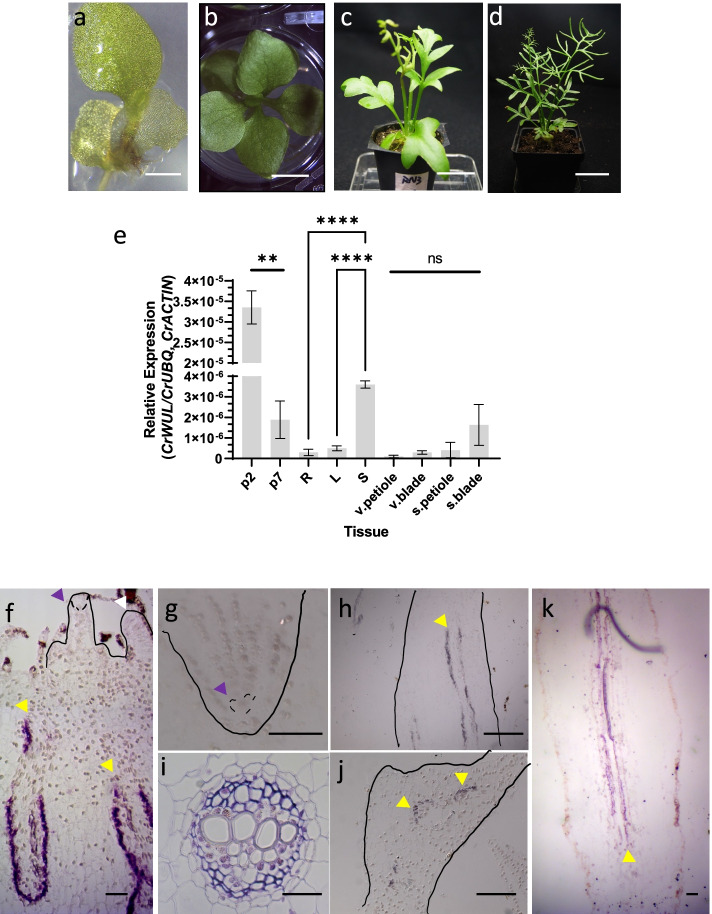
Expression of *CrWUL* in the sporophyte generation of *C. richardii.* (**a-d**) Stages of wild-type sporophytes used for expression assays; sporophyte with one leaf (*p*2) (**a**), six leaves (*p*7) (**b**), 13 expanded leaves (p13) (**c**), and mature sporophyte with sporophyll (**d**). (**e**)Expression of *CrWUL* in sporophyte tissues measured by RT-qPCR (mean ± SEM;**, *P* ≤ 0.01; ****, *P* ≤ 0.0001; *N* = 3): p2 (the whole plant), p7 (the whole plant), L (oldest fully expanded leaf), R (root), S (shoot) from a liquid grown p13 plant; v.petiole ( vegetative petiole), v.blade (vegetative leaf blade), s.petiole (sporophyll petiole), s.blade (sporophyll blade) from a mature sporophyte shown in (**d**). (**f-k**) In situ hybridization of sectioned sporophyte tissues; SAM (f), RAM (**g**), young petiole (**h**); cross section of vegetative petiole (**i**), emerging leaf blade (**j**), and root vascular tissues (**k**). White arrowhead points to leaf primordium. Purple arrowheads denote shoot (**f**) and root (**g**) apical cells, respectively. Yellow arrowheads indicate phloem tissues. Scale bars = 1 mm (**a**), 4 mm (**b**), 25 mm (**c**), 46 mm (**d**), 0.05 mm (f-k)

To examine *CrWUL* expression in different tissues and developmental stages, we took samples from sporophytes ranging from individuals with one expanded leaf to mature sporophytes with sporophylls (Fig. 2a-d) for use in qPCR analysis. Expression was the highest in p2 sporophyte (whole plant including first leaf, leaf primordia, and the first root) (Fig. 2a, e), then decreased dramatically in the p7 sporophyte (whole plant including 6 leaves, leaf primordia, and roots) (Fig. 2b, e). Among isolated roots, leaves, and shoots from p13 plants, expression is highest in the shoot tissues which contain leaf and root primordia (fern root primordia are next to leaf primordia, both are near the SAM) along with the petiole base of more mature leaves (Fig. 2e and Additional File 1: Fig S3). Sporophytes were moved to soil for the remainder of development, where they continue to generate vegetative leaves until sporophyll production (Fig. 2c, d). Expression is low in the last vegetative leaf before the sporophylls but higher in the first fully expanded sporophyll of sexually mature sporophytes (Figs. 2d, e). In situ hybridization was used to localize *CrWUL* expression. *CrWUL* expression is absent from the apical cell and core region of the SAM, developing leaf primordia (Fig. 2f), and developing root primordia including the root apical cells (Fig. 2g). In contrast, expression of *CrWUL* is detected in the longitudinal section of the shoot and leaf petiole as two long bands on either side of xylem tissues (Fig. 2f, h) and in the cross section of the vascular bundle between the pericycle and xylem, containing phloem plus any residual procambium cells (Fig. 2i). Expression continues up the petiole vascular bundles into developing leaf blades (Fig. 2j). In addition, expression of *CrWUL* is present within root vascular bundles (Fig. 2k). All sense probe control images are provided in Additional File 1: Fig S4. In conclusion, *CrWUL* is specifically expressed in phloem and is conspicuously absent from the SAM and leaf primordia.

### Phenotype of *CrWUL *RNAi knockdown lines (*crwul*)

**Fig. 3:**
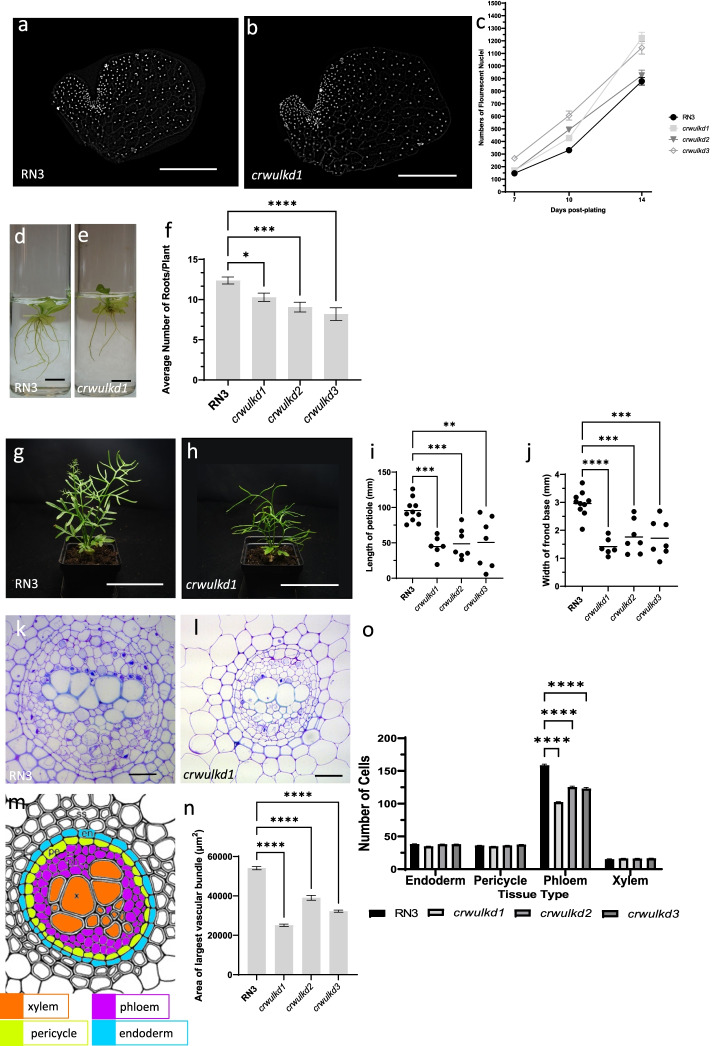
Phenotype of *crwul* knockdown lines. (**a, b**) Fluorescent images of 10-days post plating (dpp) gametophytes stained with Hoechst dye. (**c**) Numbers of florescent nuclei 7-, 10-, and 14-dpp of wild-type and *crwul* knockdown lines (*N* ≥ 19, statistics in Additional file 3: Table S2). (**d, e**) Images of wild-type and a representative *crwul* knockdown line grown for 2 weeks in liquid culture. (**f**) Numbers of roots per plant (mean ± SEM; *, *P* ≤ 0.05; **, *P* ≤ 0.01; ***, *P* ≤ 0.001; ****, *P* ≤ 0.0001 *N* ≥ 16). (**g, h**) Images of mature wild-type and a *crwul* knockdown line each has produced three sporophylls. (**i**) Length of sporophyll petiole from base to first pinna (individual lengths and mean, **, *P* ≤ 0.01; ***, *P* ≤ 0.001, *N* ≥ 6). (**j**) Width of sporophyll base (individual lengths and mean, **8, *P* ≤ 0.001; ****, *P* ≤ 0.0001, *N* ≥ 6). (k, l) Cross sections of the largest vascular bundles in the first sporophyll from wild-type and a representative *crwul* line. (m) Diagram of *C. richardii* vascular bundle with tissue types color coded; en, endoderm; pe, pericycle; ph, phloem; x, xylem. (adapted from [25]). (*n*) Area of bundles (mean ± SEM, ****,*P* < 0.0001 *N* ≥ 9) (o) Numbers of cells per section from each tissue type present in the vascular bundles (mean ± SEM; ****, *P* ≤ 0.001 *N* ≥ 10). Scale bars = 0.05 mm (a,b), 10 mm (d, e), 90 mm (g, h) and 0.05 mm (k, l)

*WOX* genes are involved in promoting cell division and growth of plant tissues containing meristems. The overall size and morphology of *crwul* knockdown lines are similar or larger than wild-type gametophytes (Fig. 3a-c). Numbers of cells in the hermaphrodite thallus increases from 7-dpp to 14-dpp and *crwul* knockdown lines remained at or above wild-type levels during development (Fig. 3c). In addition, *Ceratopteris Histone H4* (*CrH4*) expression was used as marker for cell divisions and when measured, *CrH4* expression remained at or above wild-type levels in *crwul* knockdown gametophytes (Additional File 1: Fig. S5). Knockdown of endogenous *CrWUL* transcripts in *crwul* lines was measured in 14-dpp gametophytes (Additional File 1: Fig. S6). Reduction of *CrWUL* transcripts was highest in line *crwul1* at < 7% of wild-type levels. Remaining lines averaged 30%-70% reduction in *CrWUL* transcripts. Selected gametophytes were selfed and grown in liquid for two weeks until sporophytes reached p7-8. Liquid grown sporophytes were then imaged and adventitious roots quantified (Fig. 3d-f). All *Ceratopteris* roots are shoot-borne, therefore they are termed either shoot-borne roots or adventitious [24]. *crwul* lines produced fewer numbers of roots than the wild-type (Fig. 3d, e). On average, *crwul* lines produced 9.1 roots per plant while wild-type plants produced 12.4 roots per plant, a reduction of 27% (Fig. 3f). *crwul* knockdown lines are of shorter stature due to shorter leaf petioles (Fig. 3g-j). For quantification, the petiole of the 1^st^ sporophyll of a sporophyte that had produced three sporophylls was measured and overall, petiole length decreased ~ 49% in knockdown lines and width decreased ~ 45% compared with wild-type petioles (Fig. 3i, j). Because expression of *CrWUL* is absent from the shoot and root meristems but seen in vascular bundles, using anatomical features we counted cells of various tissue types within cross sections of the 1^st^ sporophyll petiole of a sporophyte that had produced three sporophylls (Fig. 3g, h). Ceratopteris produce concentric amphicribral vascular bundles within petioles. During development *Ceratopteris* petioles increase in width and number of vascular bundles. The 13^th^ vegetative leaf of *Ceratopteris* contain three vascular bundles, while the 1^st^ sporophyll after three sporophyll produced contain five vascular bundles (data not shown). *Ceratopteris* sporophyll petioles used for quantification, either from wild-type or *crwul* lines, each contained five vascular bundles, signifying equivalent developmental time points between lines. A ring of endodermal cells encloses each vascular bundle, in which a central band of xylem is surrounded by parenchyma comprising phloem and any residual procambium, together are referred as phloem from hereon (Fig. 3k-n) [25]. All tissue types were present in the *crwul* lines but the area of the vascular bundles cross sections decreased (Fig. 3n). Generally, vascular bundles are smaller in younger petioles, therefore we quantified cell number in vascular bundles between equivalent leaves to assess whether the decreased area of *crwul* vascular bundles was associated with reduced numbers of a specific cell type. Within the *crwul* lines all tissues except phloem contained similar numbers of cells as wild-type plants (Fig. 3k-o). Phloem cell numbers, on average, were reduced by 27% in *crwul* lines when compared to wild-type (Fig. 3o). Interestingly, expression of *CrH4* decreased in p7-8 *crwul* whole sporophytes and was also expressed preferentially in the phloem of vascular bundles (Additional File 1: Fig. S5). While the overall stature of the *crwul* lines were visibly shorter than that of the wildtype, they produced the same number of fronds as the wild-type plants (data not shown). These results showed that knocking down *crwul* expression caused a decrease of phloem cell numbers, petiole length and width and production of fewer roots but not fewer leaves.

### *CrWUL* restores cambiam layer in *atwox4* null mutants

**Fig. 4:**
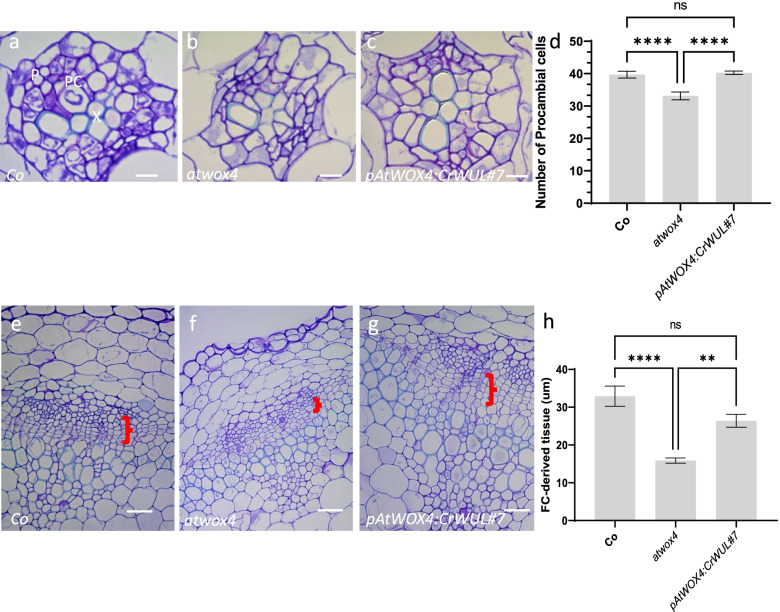
*CrWUL* restores cambium layer in *atwox4* null mutant lines. **a-c** Cross section of wild-type Col (**a**), *atwox4* null mutant (**b**) and *pAtWOX4:CrWUL* (**c**) hypocotyl. **d** Number of procambium cells per section (mean ± SEM; ****, *P* ≤ 0.0001, *N* ≥ 10). **e–g** Cross section of wild-type Col (**e**), *atwox4* (**f**) and *pAtWOX4:CrWUL* (**g**) base of 15 cm primary inflorescence. Red brackets indicate cambium cell layer (**e**–**g**). **h** Length of cambium tissues per section in base of 15 cm primary inflorescences (mean ± SEM; **, *P* ≤ 0.01; ****, *P* ≤ 0.0001, *N* ≥ 10). p: Phloem, x: Xylem, pc: Procambium. Scale Bars = 0.005 mm (a-c), 0.03 mm (**e–g**)

*AtWOX4* functions in the cambium of *Arabidopsis* hypocotyl and stem vascular tissues to maintain the number of cambium cells [14, 15]. Therefore, we asked whether the function of *WOX* genes in vascular tissues was conserved across ferns and angiosperms by expressing *CrWUL* under the *AtWOX4* endogenous promoter in an *atwox4* null mutant (Additional File 1: Fig. S7). This previously characterized T-DNA insertion mutation was chosen because it only affects vascular tissue cell proliferation but not organization [14, 15]. Three transgenic lines were selected and grown for genotyping (Additional File 2: Fig. S8) and phenotyping. *atwox4* null mutant plants used in this study have less cambium but no observable phenotype [15]. As expected, *atwox4* plants expressing *CrWUL* are indistiguishable from the wild-type and *atwox4* null mutant (Additional File 2: Fig. S9). *Arabidopsis* hypocotyl cross sections revealed that the organization of the vasculature remain the same in the wild type, *atwox4,* and *pAtWOX4:CrWUL* lines, with xylem and phloem separated by cambium (Fig. 4a-c). There were 20% fewer cambium cells produced by *atwox4* null mutants than those in the wildtype and *pAtWOX4:CrWUL* lines. *pAtWOX4:CrWUL* completely restored the procambium cell numbers in the mutant lines (Fig. 4d). Cross sections of the bottom 1 cm of 15 cm-tall inflorescence stems were examined for the production of vascular tissue (Fig. 4e-h). In wild-type stems, cells of the vascular cambium divided periclinally to produce phloem externally and xylem internally, giving rise to files of cells of each tissue (Fig. 4e). *atwox4* plants produced fewer cambial derivatives than Col and *pAtWOX4:CrWUL*, and therefore the phloem and xylem were separated by a 52% and 40% shorter distance, respectively (Fig. 4f). *pAtWOX4:CrWUL* plants restored the number of cambial cells and the distance between the phloem and xylem increased to levels measured in wild-type plants (Fig. 4g, h).

### The *S. kraussiana T3WOX/*modern gene *SkWOX11C* is specifically expressed in lycophyte vascular bundles

**Fig. 5:**
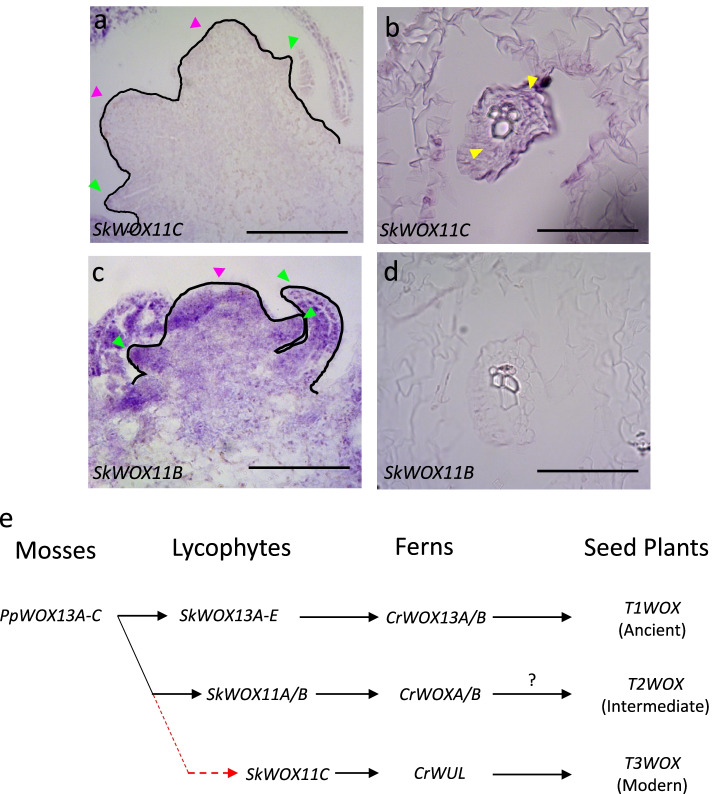
The *T3WOX/*modern *Selaginella kraussiana WOX11C* is expressed in lycophyte vascular bundles. **a** In situ of *SkWOX11C* in the shoot apex. **b** In situ of *SkWOX11C* on cross section of vascular bundle in stem tissues. **c** Expression of *SkWOX11B* in the shoot apex. **d** In situ of *SkWOX11B* on cross section of stem. **e** Abbreviated land plant phylogeny, and (**f**) Revised trajectory of *WOX* gene function in land plants (adapted from [18]). Pink and green arrows indicate shoot apical meristem and emerging leaf primordia, respectively. Yellow arrows indicate phloem. Scale bars = 0.05 mm

**Fig. 6:**
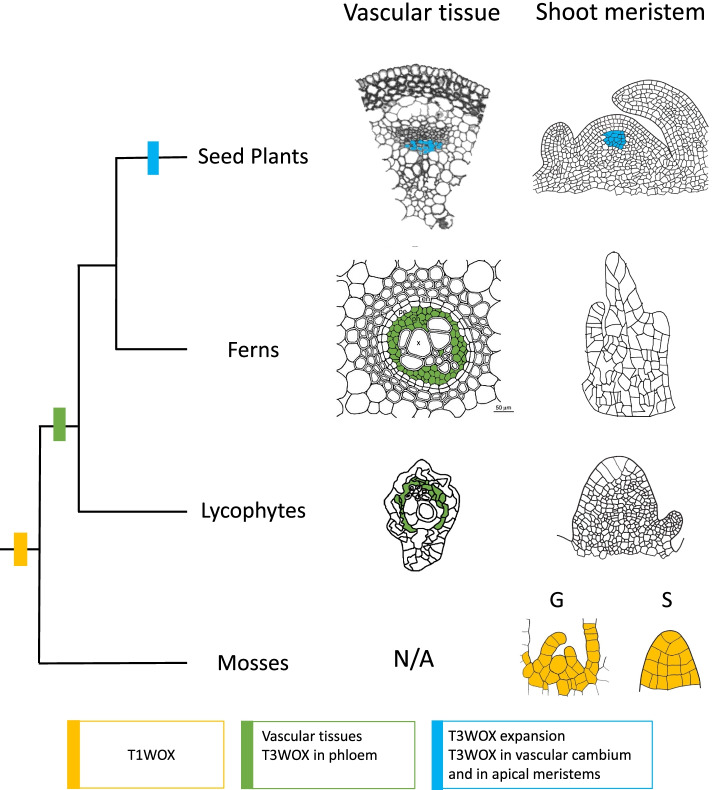
Evo/Devo of *T3WOX/*modern transciption factors. Abbreviated land plant phylogeny depicting the emergence of the *T1WOX/*ancient clade (yellow box), *T3WOX/*modern clade (green box), and *T3WOX/*modern expansion in the seed plant lineage (blue box). Expression of the *T1WOX/*ancient clade in *P. patens* is present in both the gametophore apex (G) and the sporophyte apex (S) (yellow cells). Expression of the single *T3WOX/*modern genes in *S. kraussiana* and *C. richardii* are present in the phloem (green cells) but not the SAM. After *T3WOX/*modern expansion, members of *T3WOX/*modern genes in seed plants are expressed in vascular tissues and SAM (blue cells)

Lycophytes represent the earliest extant vascular plants possessing the *T3WOX/*modern clade. Therefore, it was of interest to determine whether this gene is expressed in the vascular tissue or in the meristem regions of the lycophyte. Previous expression analysis used RNAseq of RNA samples taken from shoot tip, root tip, microphyll, and stem [26], each likely to include vascular tissue. In order to distinguish whether expression is specific to the SAM apex or to the vasculature present in all tissues, we used in situ hybridization to localize *SkWOX11C* (Fig. 5a,b). We found that expression of *SkWOX11C* is absent from the shoot apex and youngest leaf primordia of *S. kraussiana* (Fig. 5a) but is present in the vascular bundles of the shoot (not shown) and stem, specifically in the phloem, not the xylem (Fig. 5b). Interestingly, *SkWOX11B,* which is not grouped with *T3WOX/*modern genes (Additional File 1: Fig. S1), is expressed throughout the *S. kraussiana* shoot apex including leaf primordia (Fig. 5c) but is absent from vascular tissues (Fig. 5d). Sense probe controls are provided in Additional File 2: Fig. S10. These results support that the *AtWOX4-like* function may have already existed in the lycophytes. Accordingly, we revised a previously proposed trajectory showing conservation of *WOX* gene function in land plants [18] by moving *SkWOX11C* from the T2WOX/intermediate group to the T3WOX/modern clade (Fig. 5e). These results support the existence of the T3WOX/modern clade in the last common ancestor of *S. kraussiana* and ferns, as well as the recruitment of the *T3WOX/*modern genes to function in apical meristems after the divergence of ferns and seed plants (Fig. 6).

## Discussion

The WOX gene family of homeodomain transcription factors is important in maintaining the identity of stem cells and proliferation of other pluripotent cells in land plants. The T3WOX/modern clade only exists in vascular plants [16, 17]. The earliest known *T3WOX/*modern clade gene that contains the signature WUS box has been found in *Ceratopteris (CrWUL),* and the T3WOX/modern clade has subsequently expanded in seed plants. Among the many T3WOX/modern clade members in *Arabidopsis*, *AtWUL*, *AtWOX5*, and *AtWOX4* are involved in stem cell maintenance of the SAM, RAM, and VC, respectively [9, 12, 27]. Although ferns are sister to seed plants, they contain a SAM architecture which has only a single apical cell (reviewed by [28, 29]) and shoot-borne roots [30]. Initials for each leaf-root pair arise near each other, in proximity to the apical cell of the SAM. Both leaf and root grow acropetally and all the cells in each leaf and root are descendants of the leaf and root initials, respectively [24, 31]. Here we found the major site of function for CrWUL is the sporophyte vasculature, specifically the phloem. *CrWUL* expression levels affect the number of roots, but not leaves, produced. The expression of *CrWUL* was also observed in the gametophytes, where it negatively regulates cell numbers.

### *CrWUL* negatively regulates cell number in the gametophyte

*CrWUL* is widely expressed in immature hermaphroditic gametophytes, but expression declines once gametophytes reach sexual maturity (Fig. 1). The expression pattern in immature hermaphrodites is different from that of *CrWOXB*, which is more prominent in the notch region [32]. In males, the expression of *CrWUL* is not detected but *CrWOXB* is detected in undifferentiated cells, suggesting a hermaphrodite-specific role for CrWUL. Unlike *CrWOXB*, which is involved in cell division at the notch region [32], knocking down the expression of *CrWUL* has a positive effect on the cell number of the gametophytes (Fig. 3). This suggest that CrWUL negatively regulates cell division in the gametophyte, a function opposed to that of CrWOXB. Negative regulation by CrWUL may be attributed to the presence of WUS-box and to a lesser extent, the EAR domain, which is important for AtWUS nuclear export and cytoplasmic stability [33–35], both domains are absent from CrWOXB*.* In addition, Arabidopsis shoot size is inversely proportional to AtWUS concentration [36]. Perhaps CrWUL dosage and/or negative regulation of certain cell-cycle genes by CrWUL contribute to the larger gametophyte, whereas *CrWOXB* does not [32]. In *Arabidopsis*, only one *T3WOX*/modern clade gene, *AtWOX2,* is expressed in the female gametophyte in addition to sporophyte, but the function in the gametophyte is unknown [37]. Interestingly, our results show that *CrWUL* played opposite role in regulating cell division in the two generations of Ceratopteris. This may not be surprising as AtWUS is known as a bifunctional transcription factor [34]. In addition, many transcription factors change their regulatory functions in a tissue specific manner (reviewed in [38]) and/or contain dual roles (positive and negative) in either a “context-dependent” or “signal-dependent” fashion (reviewed in[39]). In sum, *CrWUL* is expressed in both generations, providing evidence that the *T3WOX/*modern clade gene may have an ancestral role of negative regulation that was co-opted from the gametophyte to the sporophyte before the divergence of ferns.

### A role for *CrWUL* in fern root and shoot development

Once specified, the *Ceratopteris* root initial (RI) undergoes four asymmetric cell divisions, resulting in four merophytes that each share a cutting face with the tetrahedral RI [30]. *CrWUL* is transiently expressed in the three proximal merophytes but never in the RI or the distal merophyte, which is destined for root-cap fate [16]. The observation that the *crwul* knockdown lines had fewer roots is consistent with its transient expression in the three proximal merophytes where it maintains their pluripotency. With insufficient amount of CrWUL, merophytes arrest, decreasing root numbers (Fig. 3). Fewer roots produced in *crwul* knockdown lines is also consistent with the report that *CrWUL* is induced by auxin to promote adventitious root production [23]. Fewer roots in *crwul* knockdown lines superficially resembled that of the *CrWOXB* knockdown lines [32] but the temporal and spatial expression patterns of the two genes are very different; *CrWOXB* is expressed in the root tip region where cells proliferate, whereas *CrWUL* is not expressed in this region but is expressed in root vasculature and at the very beginning of root initiation (Fig. 2).

In contrast to fewer roots, the number of leaves was similar between *crwul* knockdown lines and wild-type plants. However, the width and length of the sporophyll petiole were both reduced in *crwul* knockdown lines. *CrWUL* is not expressed in the leaf primordia, including the apical cells, and presumably has no role in early leaf development. As such, the decrease in petiole length and width could be due to reduction in plant growth from a combination of fewer roots and decreased phloem. This is different from *CrWOXB* which is highly expressed in the leaf primordia, including apical cells, and knockdown lines which produce fewer leaves than the wild-type plants [32]. Thus, unlike the root, leaf primordia specification has not recruited the function of a *T3WOX*/modern clade gene.

### CrWUL regulates phloem cell numbers

In the sporophyte, in addition to its expression in the three direct descendants in the RI [16], we showed that *CrWUL* expresses and functions in the vasculature, specifically for maintaining proper phloem cell numbers (Fig. 3). *AtWOX4* is known to regulate cell proliferation of the procambium and cambium of *Arabidopsis* [14, 15, 26]. Although modern ferns lack secondary growth [40], when *CrWUL* was expressed under the *AtWOX*4 promoter, it restored the cambium phenotypes in an *Arabidopsis* null mutant of *AtWOX*4 (Fig. 4). Unlike *AtWOX*4, knocking down *CrWUL* expression only decreased the phloem cell number but not the xylem or endoderm cell numbers in *Ceratopteris*, suggesting CrWUL is not involved with specifying these tissues. It also could be due to a redundant function of another *CrWOX* gene. In *Arabidopsis*, a *T1WOX*/ancient clade gene, *AtWOX14,* acts redundantly with *AtWOX4* to regulate vascular proliferation [41]. *Ceratopteris* has two *T1WOX/*ancient genes, *CrWOX13A* and *CrWOX13B*, both encode proteins which share additional peptide motifs with *AtWOX14* outside of the conserved homeodomain ([21], Additional File 2: Fig. S11).

### The function of *T3WOX/*modern clade gene in vasculature predates its SAM function

In contrast to *CrWOXB,* which is expressed broadly in the SAM and leaf primordia [32], the expression of *CrWUL* was absent from the SAM and leaf primordia outside of vasculature (Fig. 2). This discovery prompted us to determine if the role of the T3WOX/modern clade in vasculature predates ferns. The *T3WOX/*modern clade gene in the lycophytes have been suggested to contain the earliest T3WOX genes [17], and expression in the vasculature of *S. kraussiana* supports this proposed relationship (Fig. 5). However, *SkWOX11C* does not have a discernable WUS box (Data not shown), which is required for stem cell function [34]. This situation is not unique, as a T3WOX gene from the conifer *Araucaria rulei* also lacks the WUS-motif [17]. In contrast, *SkWOX11B*, which is not a *T3WOX/*modern clade gene, was expressed in SAM apex and young leaf primordia but not in vascular tissues, highlighting the role of *T3WOX/*modern clade genes in the VC of vascular seed-free plants (Figs. 5, 6). A closely related WOX gene *SkWOX11A* was not expressed in the lycophyte sporophyte [18] and was not investigated here. In a proposed WOX lineage (Fig. 5e), instead of grouping *SkWOX11C* with *SkWOX11A/B*, we place *SkWOX11C* outside the previous grouping, and in line with the T3WOX/modern clade lineage. This new grouping puts the emergence of *T3WOX/*modern genes in the last common ancestor of lycophytes and ferns, earlier than previous evidence suggested (Fig. 6). Furthermore, the origination of the T3WOX/modern clade coincides with the emergence of vascular tissues. It is only after the split of seed plants from ferns and subsequent expansion of the T3WOX/modern clade do *T3WOX/*modern genes function in apical meristems. Therefore, we compared the promoter regions of *CrWUL*, *AtWOX4*, and *AtWUS.* As expected, the upstream regions of *CrWUL* and *AtWOX4* promoters share more predicted motifs than those of *CrWUL* and *AtWUS* (Additional File 2: Fig. S12). This lends support for the phloem-specific regulation of the *T3WOX/*modern genes being the ancestral state. In other duplicated *T3WOX/*modern genes, such as *AtWUS*, the upstream regions of the promoter loses the transcription-factor binding sites for phloem-specific expression and gains those for apical meristem expression.

Interestingly, the rice *WOX4* gene (*OsWOX4*), in addition to functioning in the vasculature as does *AtWOX4*, also maintains stem-cell function in the rice SAM through a possible model different from that proposed for *Arabidopsis* [42]. The broad expression of *OsWOX4* in the SAM overlaps with the expression domain of a *CLE*-like gene, thus eliminating the requirement of a mobile WOX protein. The dual function of OsWOX4 in the vasculature and the stem-cell maintenance in the SAM further supports that the latter function has evolved more recently. It would be interesting to see whether the full-length CrWUL when expressed in the rice SAM can complement the OsWOX4 function in *OsWOX4* knockdown lines [42, 43]. Among *Arabidopsis T3WOX/*modern clade genes, all but *AtWOX4* can complement the *AtWUS* stem-cell maintenance function in *atwus* mutants [7], suggesting either divergent transcriptional targets and/or lack of mobility of AtWOX4 protein preventing complementation. CrWUL, when truncated, is mobile and when expressed in the OC and the QC of *Arabidopsis,* can restore the SAM and RAM functions in *atwus* and *atwox5* mutants, respectively [21], suggesting CrWUL has acquired stem-cell function and the transcriptional targets required for this function. The remaining unanswered question is which, if any, of the *CrWOX* genes are involved in maintaining apical cell identity in the fern.

In Conclusion, the results presented here support the occurrence of co-option of the *T3WOX/*modern clade gene from the gametophyte to function in vasculature and root development in the sporophyte. The function in vasculature is likely to have existed in the lycophyte T3WOX/modern clade progenitor and this function predates its SAM function found in many seed plants.

## Methods

### Plant material and growth conditions

Fern spores used in experiments are of *Ceratopteris richardii* strain RN3 (Carolina Biological Supply Company, Burlington, NC) as the wildtype. Spores of RN3 and *CrWUL* RNAi suppression lines (*crwul*) were surface sterilized with 4% sodium hypochlorite and 0.1% Tween-20 for 4 min, rinsed 4–5 times with sterile water and dark treated for 3–5 days to synchronize germination. Spores were then plated on basal media (1/2 MS salts, pH 6.0, 0.8% agar, 100 µg/mL ampicillin) and grown under humidity domes at 26 °C in 16/8 h light/dark cycle at 100 µM/m^−2^ s^−1^ under Bright white (3500 K) and Daylight (6500 K) fluorescent lights (GE Lighting, East Cleveland, OH) for gametophyte development. Basal media with spores were inverted after 10-days post-plating (10-dpp) to prevent water condensation from causing premature fertilization. For selfing, a few drops of sterile water were added to sexually mature hermaphrodite gametophytes in 24-well dishes. Wild-type and *crwul* sporophytes with 7–8 leaves (p8-p9) were moved to basal liquid media (1/2 MS salts, pH 6.0, 100 µg/mL ampicillin) to facilitate root growth and take samples for root counts. After 2 weeks in basal liquid media, sporophytes were transplanted into Pro-mix PGX (Premier Tech Horticulture, Quakertown, PA) and grown at 23 °C with 16/8 h light/dark cycle at 80 µM/m^−2^ s^−1^under fluorescent lights (see above) until sporophylls were harvested.

*Arabidopsis atwox4* null-mutant seeds (GK_462G01, N376572) [14, 15, 44] used for complementation were obtained from the Arabidopsis Biological Resource Center (ABRC, Ohio State University, Columbus, OH). Seeds were sown onto Pro-mix PGX soil with one capful of Osmocote Flower and Vegetable (The Scotts Company, Marysville, OH) added to the soil, cold treated for 3-days at 4 °C then grown under the same lighting conditions as *Ceratopteris* sporophytes (see above). *Arabidopsis atwox4* null-mutant plants were genotyped with primer sets 2 and 3 once rosette leaves were formed (See Additional File 3: Table S1 for primer sequences). Col-0 plants were confirmed absent of T-DNA insertions with primer set 1. DNA extraction was carried out as described [45]. *S. kraussiana* were purchased from miniature-gardening.com (Miniature Gardening, Winter, WI). Plants were grown in Pro-mix PGX soil in a transparent glass trough, with a glass lid to maintain humidity, under natural light at 40 µM/m^−2^ s^−1^, 23 °C and 16/8 h light/dark cycle.

### In situ hybridization

Antisense and sense RNA in situ probes were synthesized according to [32]. Templates for probe synthesis were cloned into the pENTR vector (Life Technologies, Carlsbad, CA) and sequenced at the Carver Center for Genomics or Iowa Institute of Human Genetics (University of Iowa, Iowa City, IA). From 1 µg of PCR products amplified using primers containing T7 promoter sequences (IDT, Coralville, IA), DIG-labeled RNA probes were synthesized using T7 RNA polymerase (Agilent, Santa Clara, CA) and DIG-RNA labeling mix (Roche Diagnostics, Indianapolis, IN). DIG-labeled RNA was precipitated in 2.25 M LiCl overnight at -20 °C and resuspended in ddH20. RNA probe concentration was estimated with a Nanodrop One (Thermo-Scientific, Waltham, MA) then diluted 1:1 with deionized formamide. Diluted probes were stored at -20 °C until use.

Tissues for whole mount and sectioned in situ hybridization were prepared as described previously [32]. Gametophytes were fixed in FAA (formaldehyde: ethanol: acetic acid, 3.7%:50%:5% v/v respectively) at room temperature for 1–2 h. FAA was replaced with 70% ethanol and tissues were stored at -20 °C. Sporophyte tissues from *Arabidopsis, Ceratopteris* and *S. kraussiana* were fixed in 4% paraformaldehyde in 1 × PBS under vacuum for 45 min. Fixative was replaced and tissues were incubated in fix for 1–2 days at 4 °C. Tissue dehydration, embedding, pre-hybridization, hybridization and detection were all described previously, except dehydration post-color development was omitted and slides were mounted in glycerol [32]. Embedded tissues for sectioned in situ hybridization were sectioned at 8–10 µm with a rotary microtome.

### Plant transformation

A 737 bp (region 1) or a 368 bp (region 2) (see Additional File 3: Table S1 for primer sequences) of *CrWUL* coding sequence was cloned into pH7GWIWG2(I) vector using the Gateway technology as described by [46, 47]. Constructs were introduced into *Agrobacterium tumefaciens* strain GV3101 from *E. coli* with an *E. coli* helper strain containing the pRK 2013 plasmid. Stable transformation of young *Ceratopteris* gametophytes was conducted as described previously [47]. Successfully transformed gametophytes (T_0_) were selected on media containing 5 µg/mL hygromycin, selfed and grown as described in [47]. From more than 20 independent transgenic lines isolated, 10 were chosen for qPCR analysis and phenotyping.

For complementation experiments, a 2 kb fragment upstream of the ATG start codon of *AtWOX4* was amplified with primers as described in [14] adapted with either *HindIII* or *PacI* restriction sites for cloning. The amplified fragment was cloned into a pMDC83 vector carrying *CrWUL* by replacing the 35S promoter with the *AtWOX4* 2 kb upstream sequences. Constructs were introduced into *A. tumefaciens* as described above. Transformation of *Arabidopsis atwox4* null mutant sporophytes was performed by the floral spraying method [48]. Seeds were collected and positive transformants were selected on ½ MS media with 15 µg/mL hygromycin. Resistant plants were transferred to soil before seed collection.

### RNA extraction and RT-qPCR

Tissues harvested for RNA extraction were flash frozen in liquid nitrogen then stored at -75 °C. Total RNA was extracted from frozen tissue with the Quick-RNA MiniPrep (Plus) kit (Zymo Research, Irvine, CA) and 500 ng of total RNA was used for cDNA synthesis with MMLV reverse transcriptase (New England Biolabs, Ipswich, MA) with either N9 random or oligo-dT [16] primers (IDT Coralville, IA).

For RT-qPCR, three biological and two technical replicates were performed for each time point. Total RNA extraction and cDNA synthesis is described as above. Detection of amplification was performed using PerfeCTa SYBR Green FastMix (Quantabio, Beverly, MA) with the Roche LightCycler 480 Real-Time PCR system (Roche Diagnostic, Indianapolis, IN). The PCR protocol was as follows: initial denaturing of 10 min at 95 °C, followed by 45 cycles of denaturing (10 s at 95 °C), annealing (10 s at 59 °C) and extension (20 s at 72 °C), with a single fluorescence read at the end of each extension time. Melting curve analysis was performed to verify absence of primer dimers and non-specific products. Expression was measured relative to *CrUBQ* alone or *CrUBQ* and *CrActin* using the delta Ct method [49].

### Phenotyping

The following tissues were used for observation and cell counts of vascular bundles: 1 cm from the base of the 1^st^ fully expanded sporophyll of *Ceratopteris* sporophytes with three sporophylls, 2-week-old *Arabidopsis* hypocotyl and 1 cm from the base of 15 cm tall *Arabidopsis* primary inflorescences. Tissues were embedded in Technovit 8100 (Kulzer, Hanau, Germany) resin according to user instructions with the following modifications: fixation was carried out as described above and dehydration was carried out in a graded acetone series. Resin blocks were sectioned at 1.5–1.75 µm with a glass knife on a Leica EM UC6 ultramicrotome (Leica Microsystems, Buffalo Grove, IL) at the Central Microscopy Research Facility (University of Iowa, Iowa, IA). Sections were dried and stained briefly in 0.025% (w/v) Toluidine blue O, mounted in Permount™ and imaged as described above.

For gametophyte cell counts, 7-,10- and 14- dpp gametophytes were collected and cleared in 100% EtOH overnight at 4 °C, rinsed in water and stained in Hoechst 33,342 (7-, 10-dpp: 40 µg/mL; 14-dpp: 4 µg/mL). For gametophytes aged 7–10 dpp, images were collected using a Leica stereomicroscope with a DAPI filter and a Qicam camera (Qimaging, Surrey, BC, Canada). Gametophytes 14-dpp, were captured with a Sony α35 digital camera through the ocular of a Zeiss compound light microscope with DAPI filter. Images were then processed using ImageJ (National Institute of Health, Bethesda, MD). First, images were mean filtered with a radius of 15 pixels and subtracted from the original image to reduce noise. Gametophytes were selected by outlining the thallus with a Wacom Intuos tablet (Wacom, Saitama, Japan). Processed images were then passed through an object identification pipeline in CellProfiler v3.1.9 (Broad Institute, Cambridge, MA). The pipeline used a global Otsu threshold with a smoothing scale of 1.3488, distinguished clumped objects by shape, and used a propagation method of drawing dividing lines between objects. The typical diameter of objects allowed was adjusted for each gametophyte timepoint, with a Min–Max range of 6–25 pixels and narrowed until the output no longer identified background cell wall fluorescence. The number of accepted objects was collected for each image. Pipeline is available via Github [50].

*Ceratopteris* petiole length and width measurements were performed on images of the 1^st^ fully expanded sporophyll of sporophytes with three sporophylls. The petiole width was measured 2–3 mm above the site of dissection from the plant. The length was measured from 2–3 mm above the dissection site up the petiole to the first set of pinnae. Width and length were measured using Photoshop CC (Adobe Inc., Mountain View, CA).

### Microscopy and cell counting

The images of in situ and Toluidine Blue-O-stained samples were acquired with a Zeiss Axiocam ERC 5 s digital camera on a Zeiss compound light microscope (Carl Zeiss Microscopy LLC, Thornwood, NY). To confirm gene expression patterns, each in situ experiment was repeated at least two times using different biological samples. Photoshop CC (Adobe Inc., Mountain View, CA) was used for counting cells in the vascular bundle and numbers of adventitious roots from images.

### Statistical analysis

Statistical analysis of *CrWUL* transcript abundance in *crwul* lines, vascular-bundle cell numbers in *Arabidopsis* and root numbers were conducted with one-way ANOVA, while *Ceratopteris* vascular bundle cell numbers and gametophyte cell counts were determined with two-way ANOVA with the Greenhouse–Geisser correction. For vascular bundle cell numbers, the one-way ANOVA was followed by Tukey’s multiple comparison test. For the gametophyte cell counts, a Dunnett’s multiple comparisons test was used. All calculations were done in GraphPad Prism version 9.0.0 (GraphPad Software, San Diego, CA).

### Bioinformatic analysis

Upstream sequence for promoter comparisons of *AtWUS* and *AtWOX4* were obtained from TAIR [51]. *CrWUL* upstream sequence were obtained from [23]. Sequences were compared with PlantPAN3.0 [52].

### Phylogeny of Selaginella WOX proteins

Multiple sequence alignment of full-length Selaginella WOX proteins was conducted with M-coffee [53] and Maximum-Likelihood trees were generated in MEGA X [54] with 500 bootstrap replicates. Protein sequences for *Osctreococcus tauri*, *Ostreococcus lucimarinus*, *Selaginella kraussiana* and *Selaginella moellendorffii* SmWOX13 were obtained from Phytozome [55]. *Selaginella moellendorffii* SmWOXII was obtained from Genbank [56]. Full-length protein sequences are provided in Additional File 4.

## Supplementary Information

**Additional file 1.** 

**Additional file 2.** 

**Additional file 3.** 

**Additional file 4.** 

## Data Availability

The materials, datasets used and/or analyzed during the current study are available from the corresponding author on reasonable request.

## Abbreviations

SAM: Shoot apical meristem
RAM: Root apical meristem
VC: Vascular cambium
WOX: Wuschel-related homeobox
STM: Shoot-meristemless
SHR: Shortroot
OC: Organizing center
CZ: Central zone
Dpp: Days-post-plating
qPCR: Quantitative polymerase chain reaction
WUS: Wushcel
RI: Root initials
QC: Quiescent center
MS: Murashige and Skoog
FAA: Formaldehyde ethanol acetic acid
UBQ: Ubiquitin
DAPI: 4′,6-Diamidino-2-phenylindole
NCBI: National Center for Biotechnology Information
TAIR: The Arabidopsis Information Resource
