# Supplementary material for: Vascular function of the T3/modern clade WUSCHEL-Related HOMEOBOX transcription factor genes predate apical meristem-maintenance function
Source: BMC Plant Biol. 2022 Apr 25;22:210. doi: 10.1186/s12870-022-03590-0 (PMC9036803; doi:10.1186/s12870-022-03590-0)
Supplement: Supplementary file 2 — Additional file 2. [file 12870_2022_3590_MOESM2_ESM.pdf]

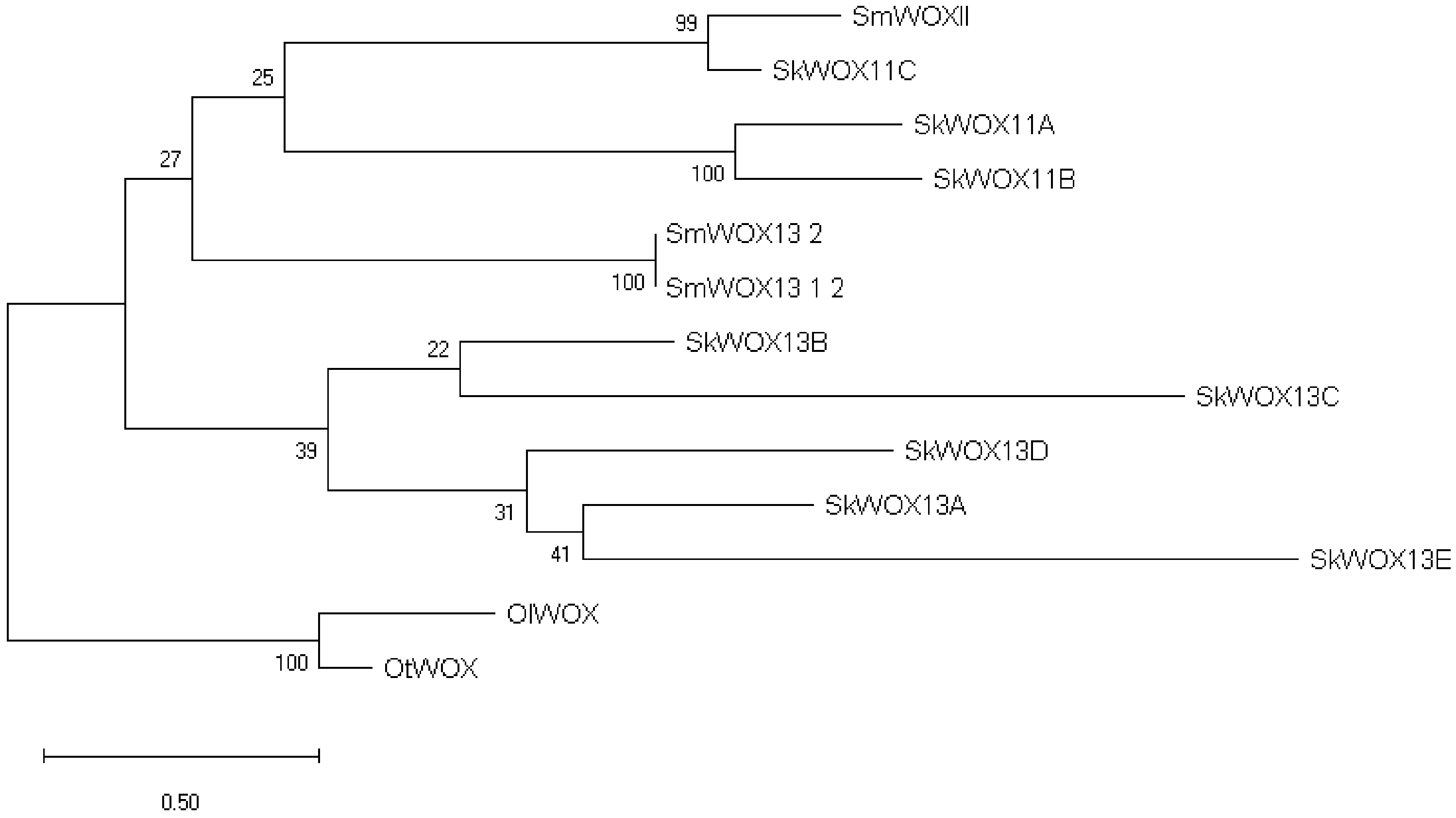

Figure S1: Phylogeny of Selaginella WOX proteins. Abbreviations *Ol*, *Osctreococcus lucimarinus*; *Ot*, *Ostreococcus tauri*; *Sm*, *Selaginella moellendorffii*; *Sk*, *Selaginella kraussiana*. Relatedness was inferred using Maximum-likelihood and 500 bootstrap replicates. Alignment of sequences was conducted with M-coffee (53) and cladogram constructed with MEGA X (54). Numbers represent percentage of trees with same grouping.

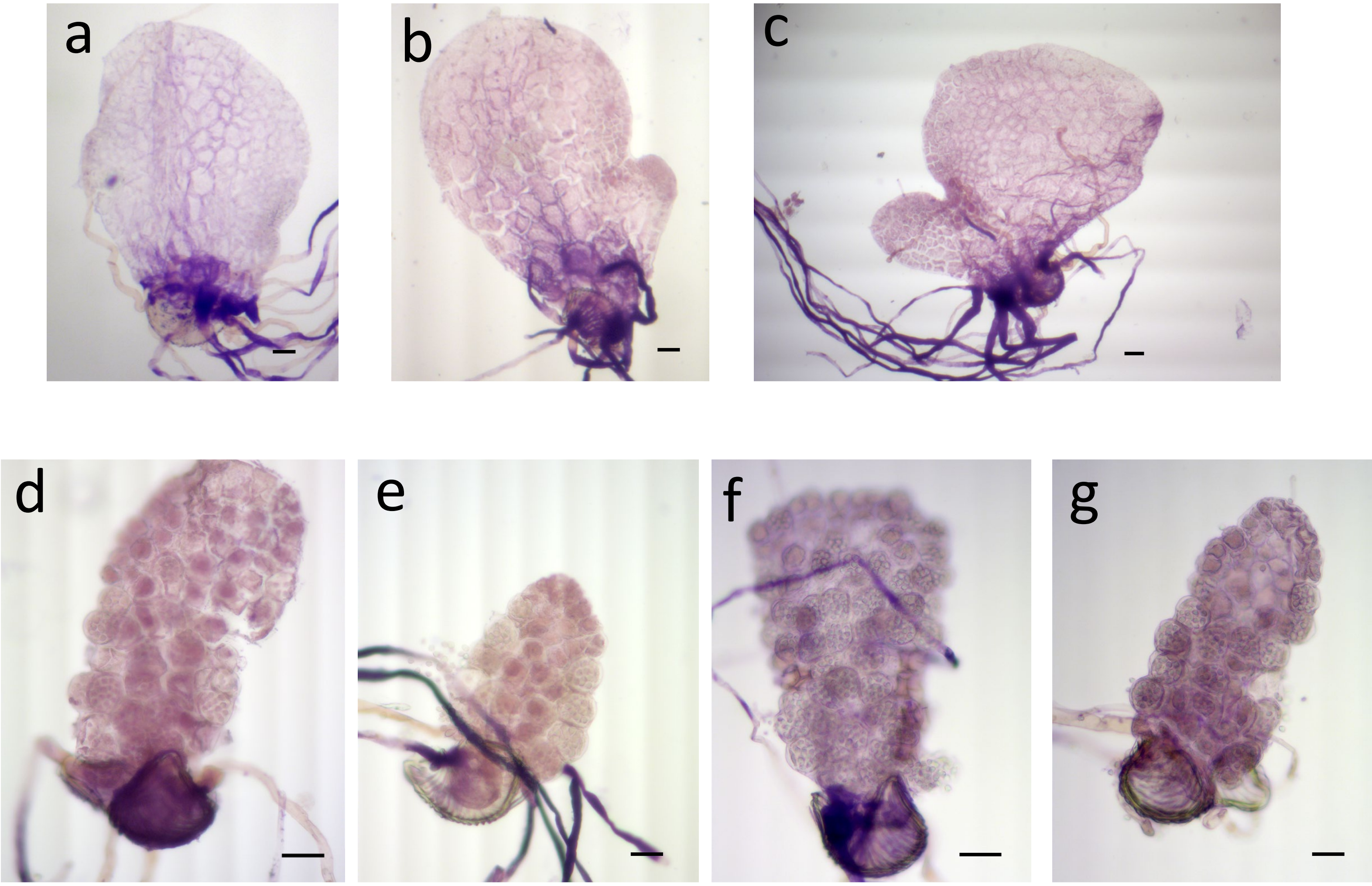

Figure S2: Whole mount *in situ* hybridization of *CrWUL* in *C. richardii* gametophytes. (a-c) Hermaphroditic gametophytes at 7-day (a), 9-day (b), and 13-day (c) old hybridized with sense probe. (d-g) Male gametophytes at 7-day (d, e) and 13-day (f,g) old hybridized with anti-sense (d, f) or sense probe (e, g). Scale bars = 0.05mm.

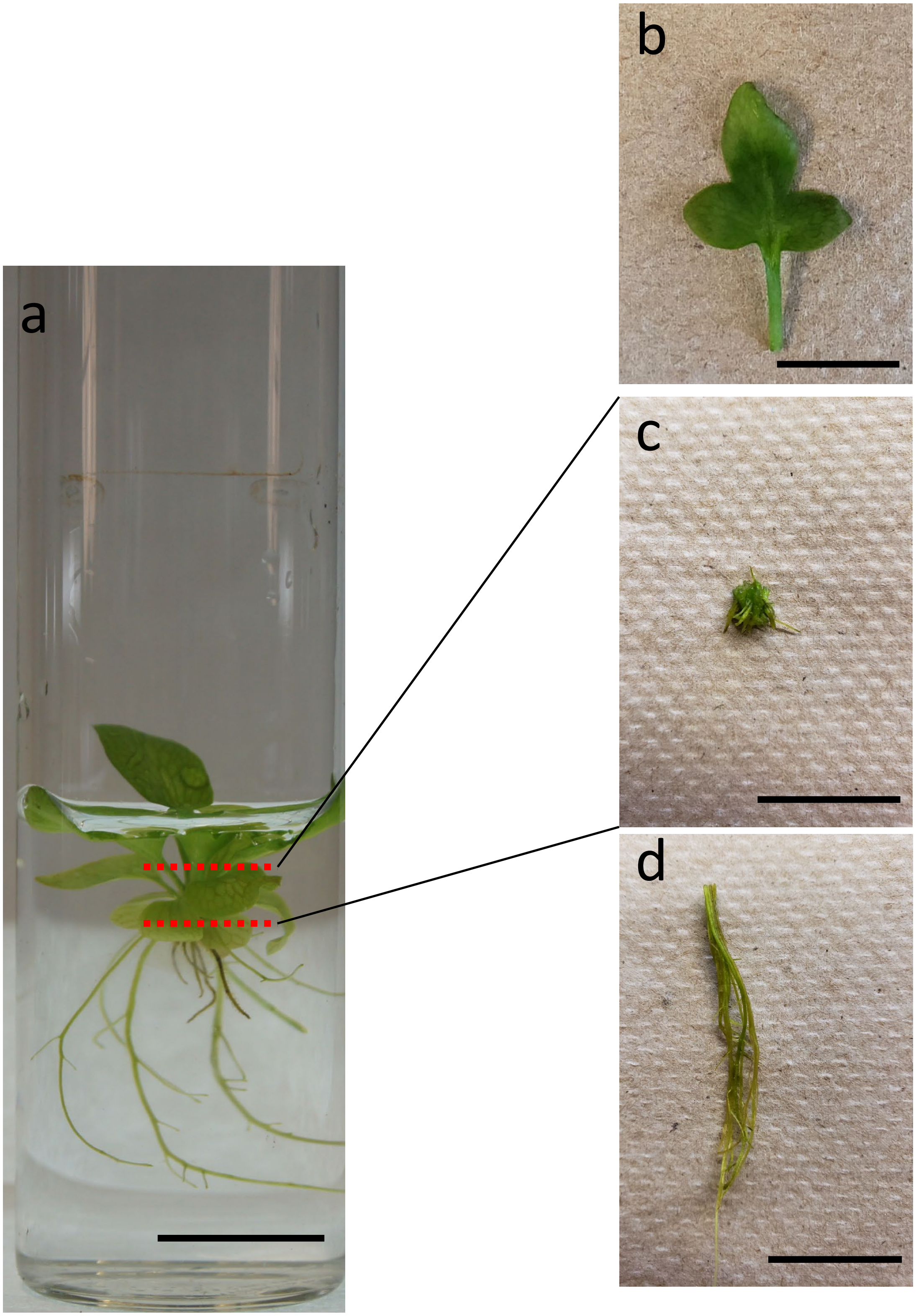

Figure S3: Dissection diagram of *C. richardii* sporophytes tissues for RT-qPCR of *CrWUL*. (a) Whole-sporophyte before dissection. (b) Single dissected leaf. (c) Shoot with leaves and roots removed. (d) Roots. Red dotted lines denote plane of dissection. Scale bars = 20µm

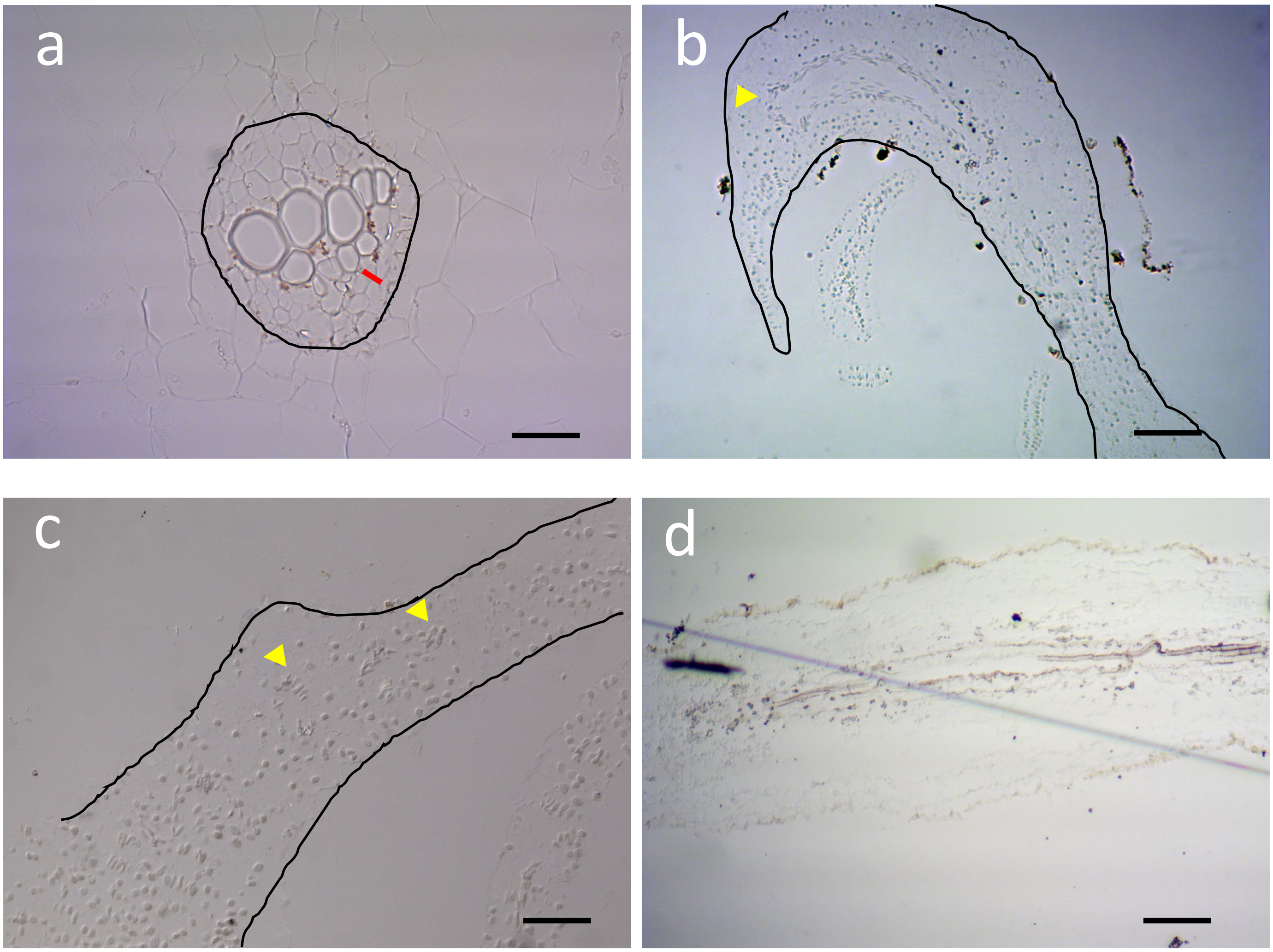

Figure S4: ***In situ*** **hybridization of** ***CrWUL*** **sense probes in** ***C. richardii*** **sporophyte tissues.** (a,b) Vascular bundles of the vegetative leaf petiole; cross-section (a), longitudinal section (b). (c) Vegetative leaf blade. (d) Root. Yellow arrowheads denote vascular bundles. Red bar signifies phloem. Scale bars = 20µm (a, c), 200µm (b, d).

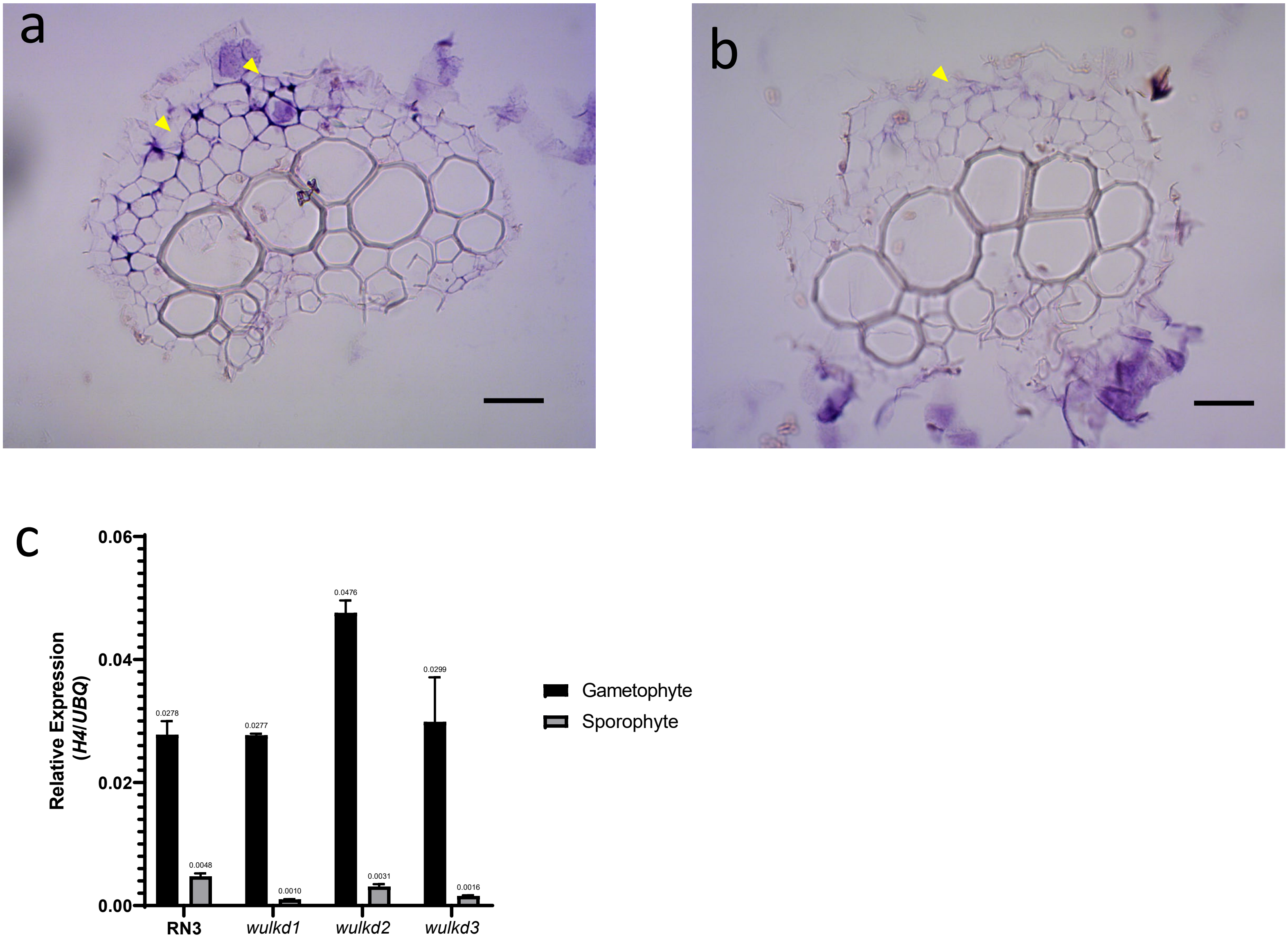

Figure S5: Expression of *CrH4* in vascular bundles and *crwul* knockdown lines. (a,b) *In situ* hybridization of *CrH4* in vascular bundles; antisense probe (a), sense probe (b). (c) Relative expression of *CrH4* in p7-8 sporophyte and 14 dpp gametophyte tissues of wild-type and *crwul* knockdown lines. Yellow arrows point to phloem. Scale bars = 20µm.

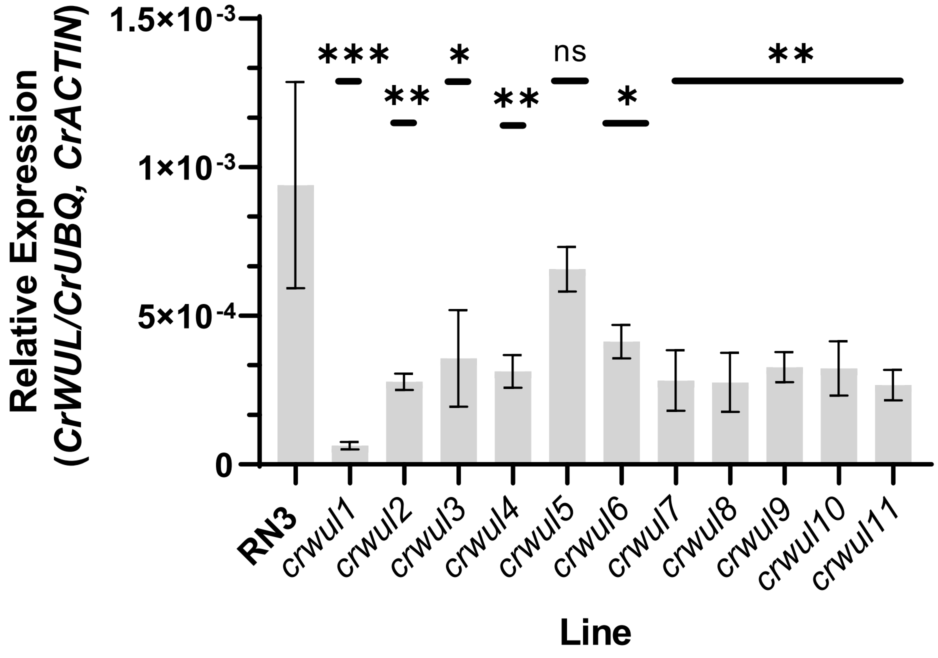

Figure S6: Expression of *CrWUL* in *crwul* RNAi knockdown lines.
CrWUL expression measured relative to *CrUBQ* and *CrACTIN* (mean ± SEM; ns, not significant; *, *P*≤0.05; **, *P*≤0.01; ***, *P*≤0.001 N≥3).

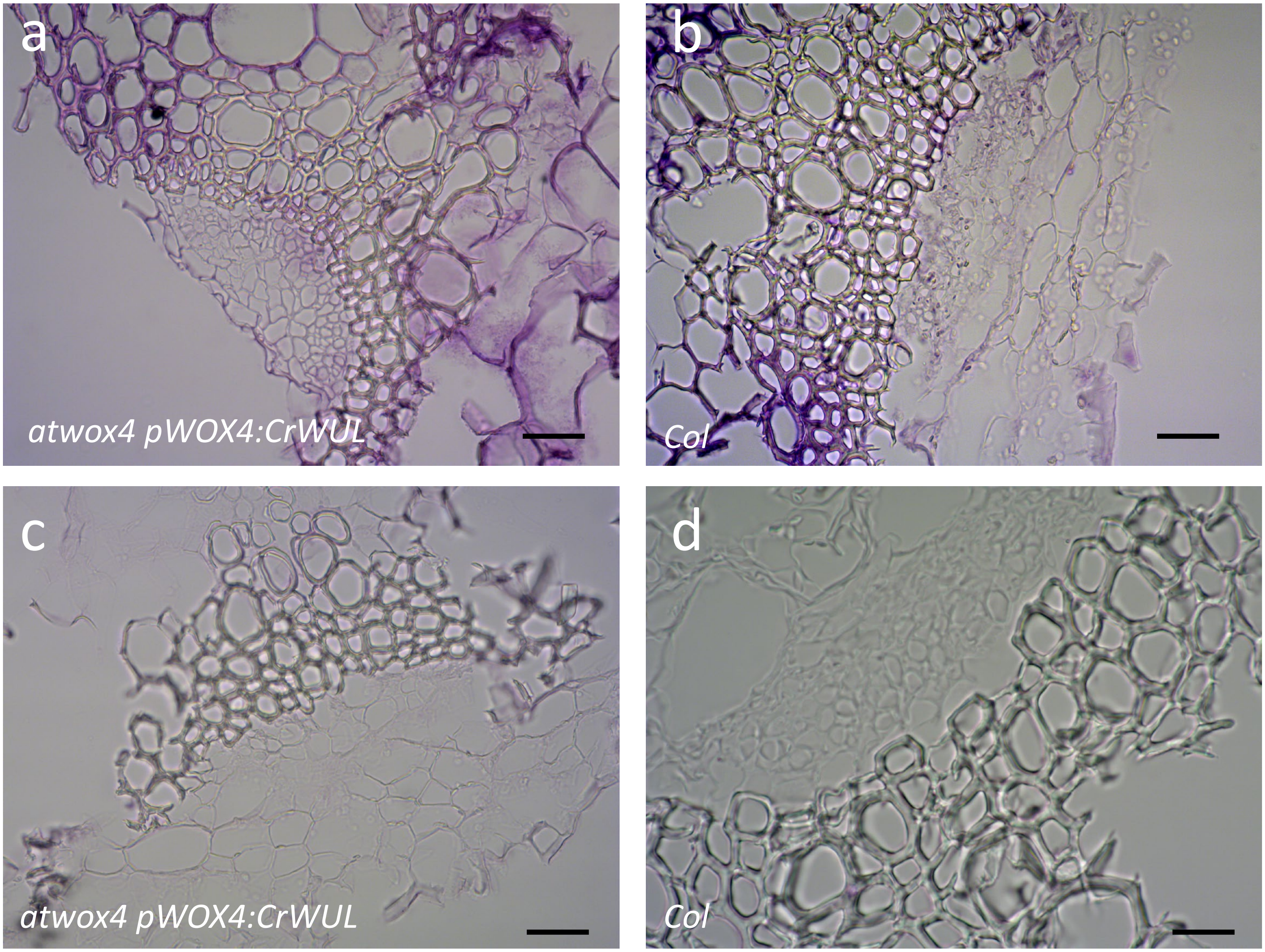

Figure S7: Expression of *CrWUL* and *AtWOX4* in vascular bundles of complemented *atwox4* null mutants and Col plants. (a) *CrWUL* anti-sense probe. (b) *AtWOX4* anti-sense probe. (c) *CrWUL* sense probe. (d) *AtWOX4* sense probe. Scale bars = 20µm.
