# Supplementary material for: Vascular function of the T3/modern clade WUSCHEL-Related HOMEOBOX transcription factor genes predate apical meristem-maintenance function
Source: BMC Plant Biol. 2022 Apr 25;22:210. doi: 10.1186/s12870-022-03590-0 (PMC9036803; doi:10.1186/s12870-022-03590-0)
Supplement: Supplementary file 3 — Additional file 3. [file 12870_2022_3590_MOESM3_ESM.pdf]

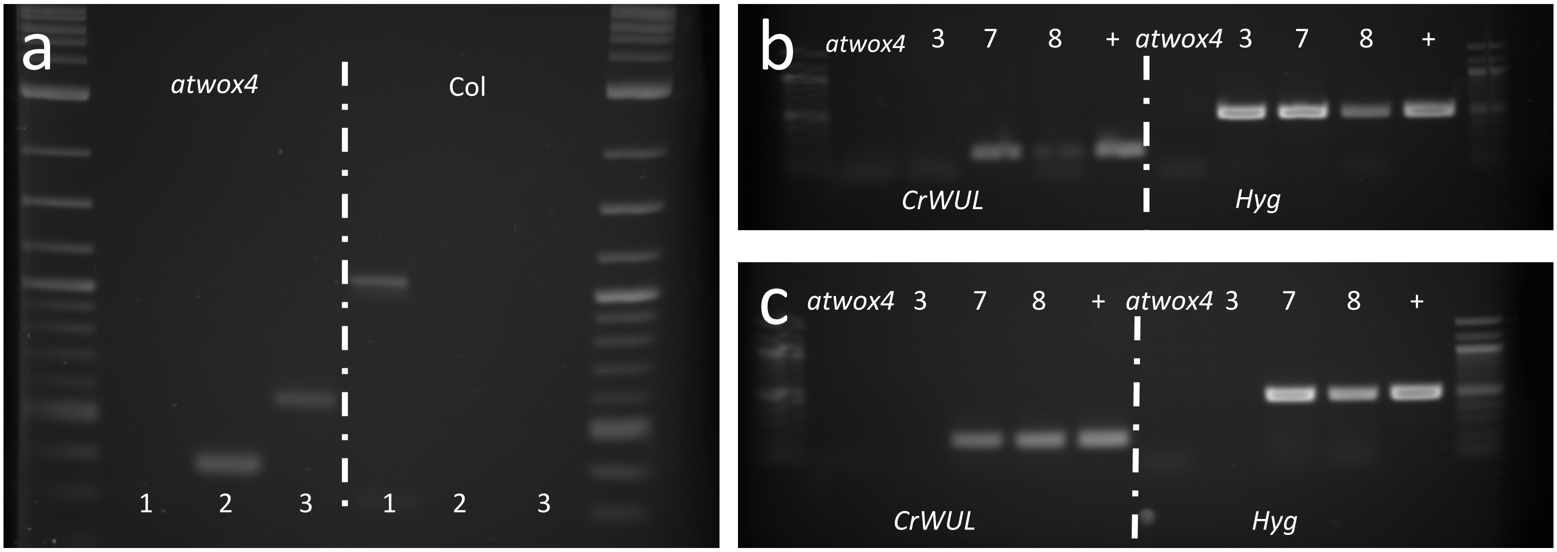

Figure S8: Genotyping *pAtWOX4:CrWUL* plants in *atwox4* null background. (a) Presence of TDNA insertion in Arabidopsis *atwox4* null-mutant seeds (GK_462G01, N376572). 1, Primer Set 1; 2, Primer Set 2; 3, Primer Set 3. Primer sequences provided in Table S1. (b) Presence of *CrWUL* and Hygromycin-resistance gene in *atwox4* and *pAtWOX4:CrWUL 3, 7* and *8* genomes. (c) Expression of *CrWUL* and Hygromycin-resistance genes in *atwox4* and *pAtWOX4:CrWUL 3, 7* and *8* transcripts. +, positive control in (b, c) is expression vector used in transformation experiments.

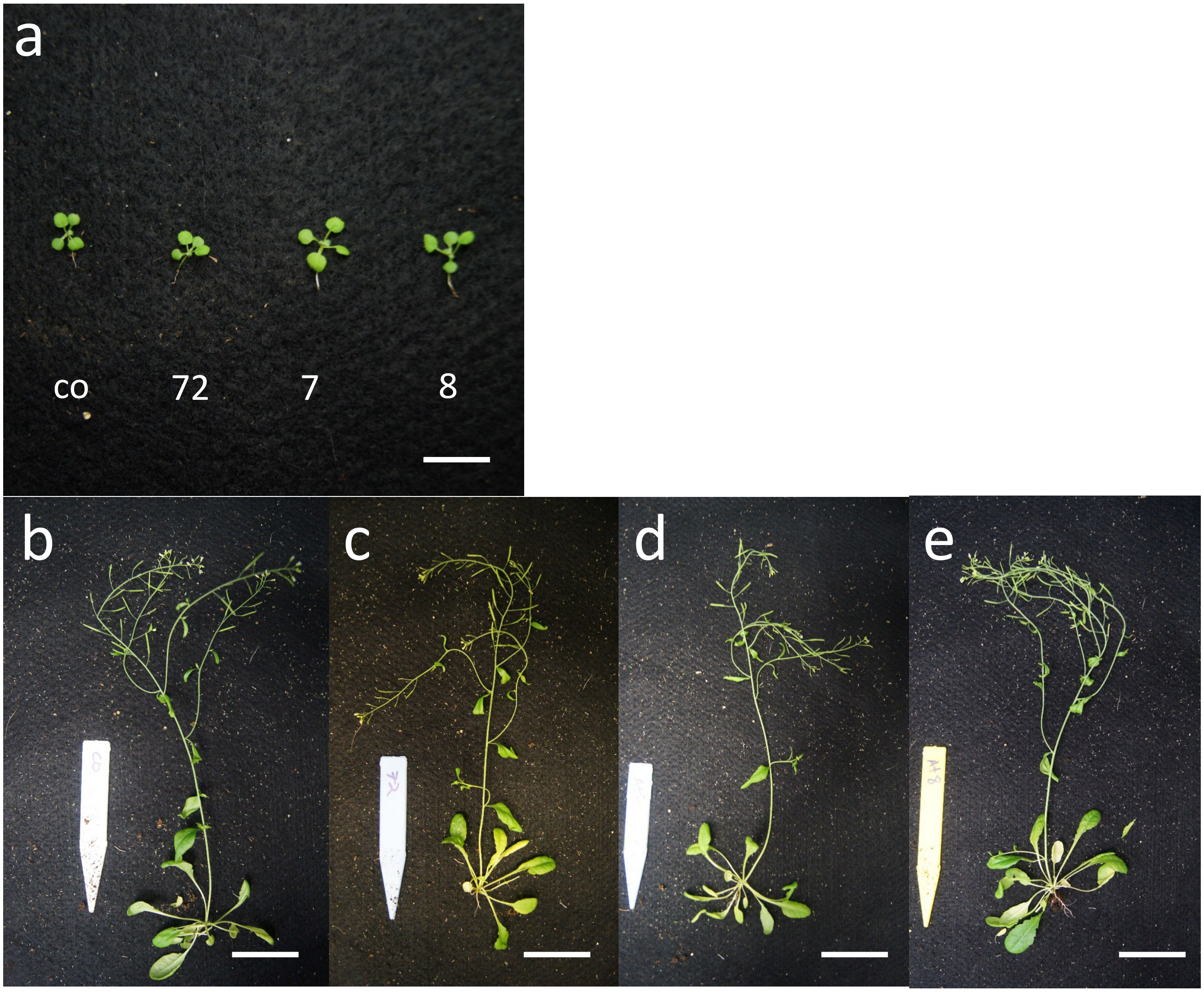

Figure S9: Whole plant morphology of *atwox4* null mutants and *pAtWOX4:CrWUL* lines. (a) Young sporophytes. (b-d) Mature sporophytes of: Col, Colombia (a,b); 72, *atwox4* null (a,c); 7, 8 are two lines of *pAtWOX4:CrWUL* (d,e). Scale bars = 2cm (a), 4cm (b-e).

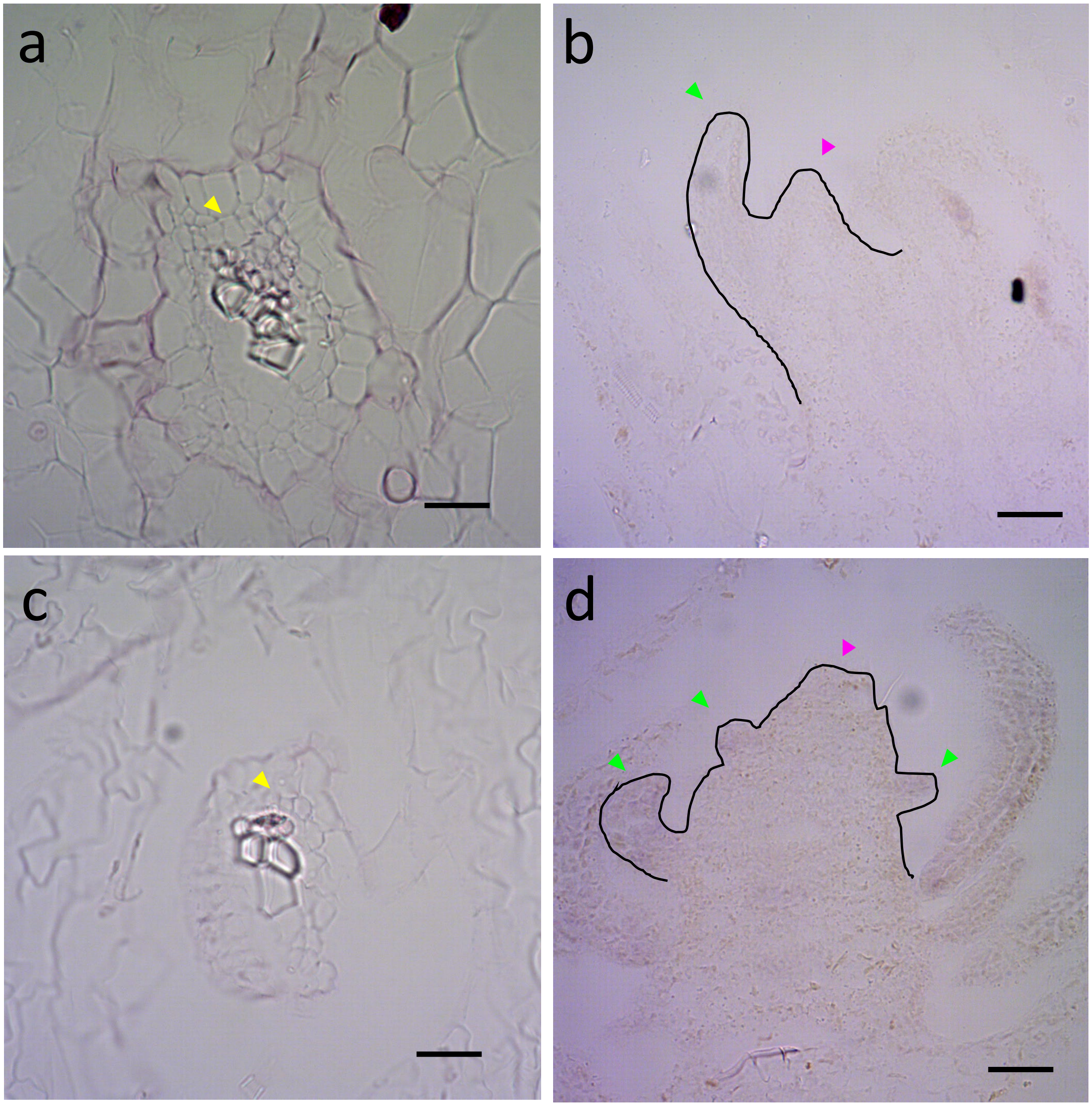

Figure S10: ***In situ*** **hybridization of sense probes in** ***S. kraussinia*** **sporophyte tissues.** (a,b) *SkWOX11C*; vascular bundles (a), shoot (b). (c, d) SkWOX11B; vascular bundles (c), shoot (d). Green and purple arrows indicate leaf primordia and shoot, respectively. Yellow arrows denote phloem. Scale bars = 20µm (a, c), 100µm (c,d).

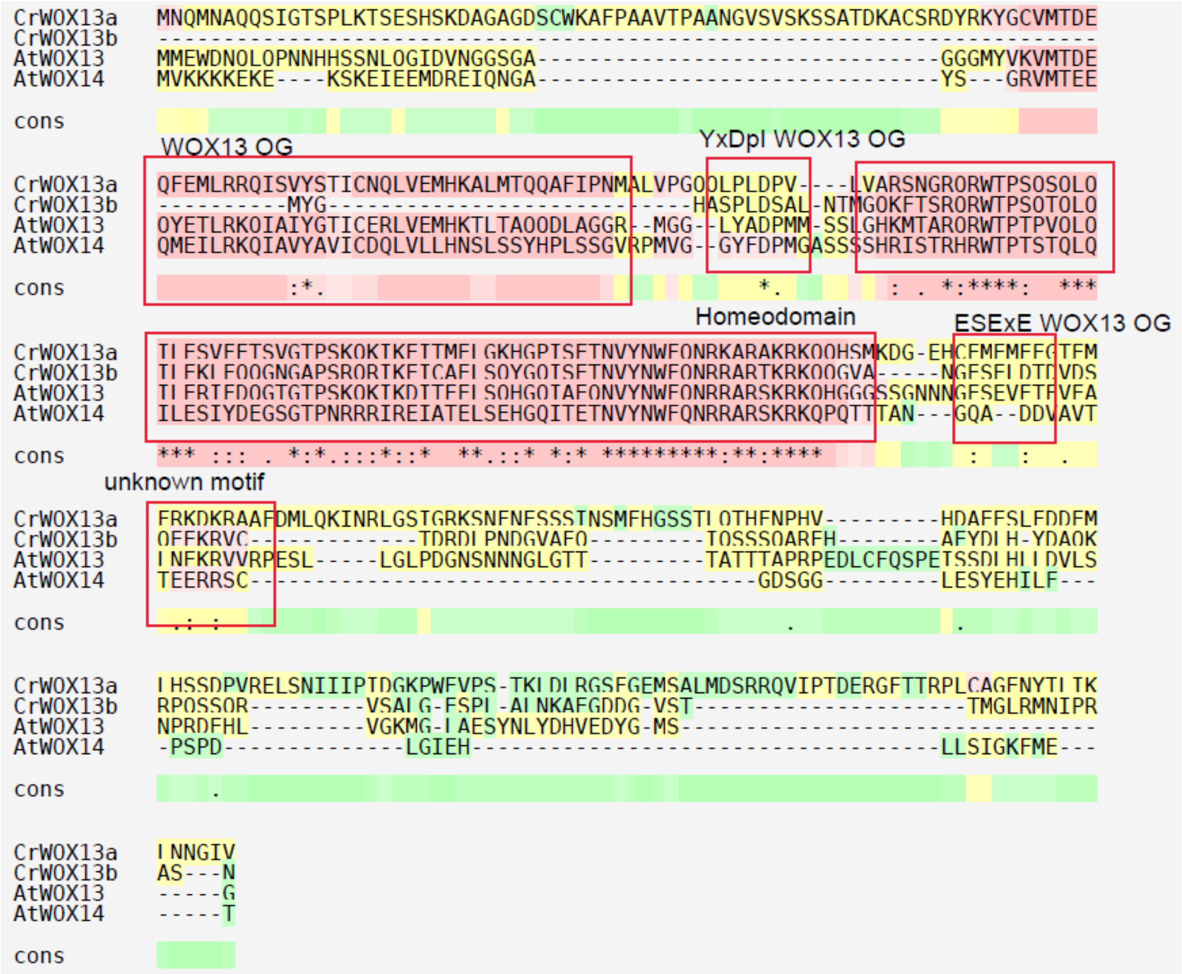

Figure S11: Alignment of *C. richardii* and *A. thaliana* T1WOX/ancient clade. Full length T1WOX/ancient clade sequences with known orthologous groups named and outlined.

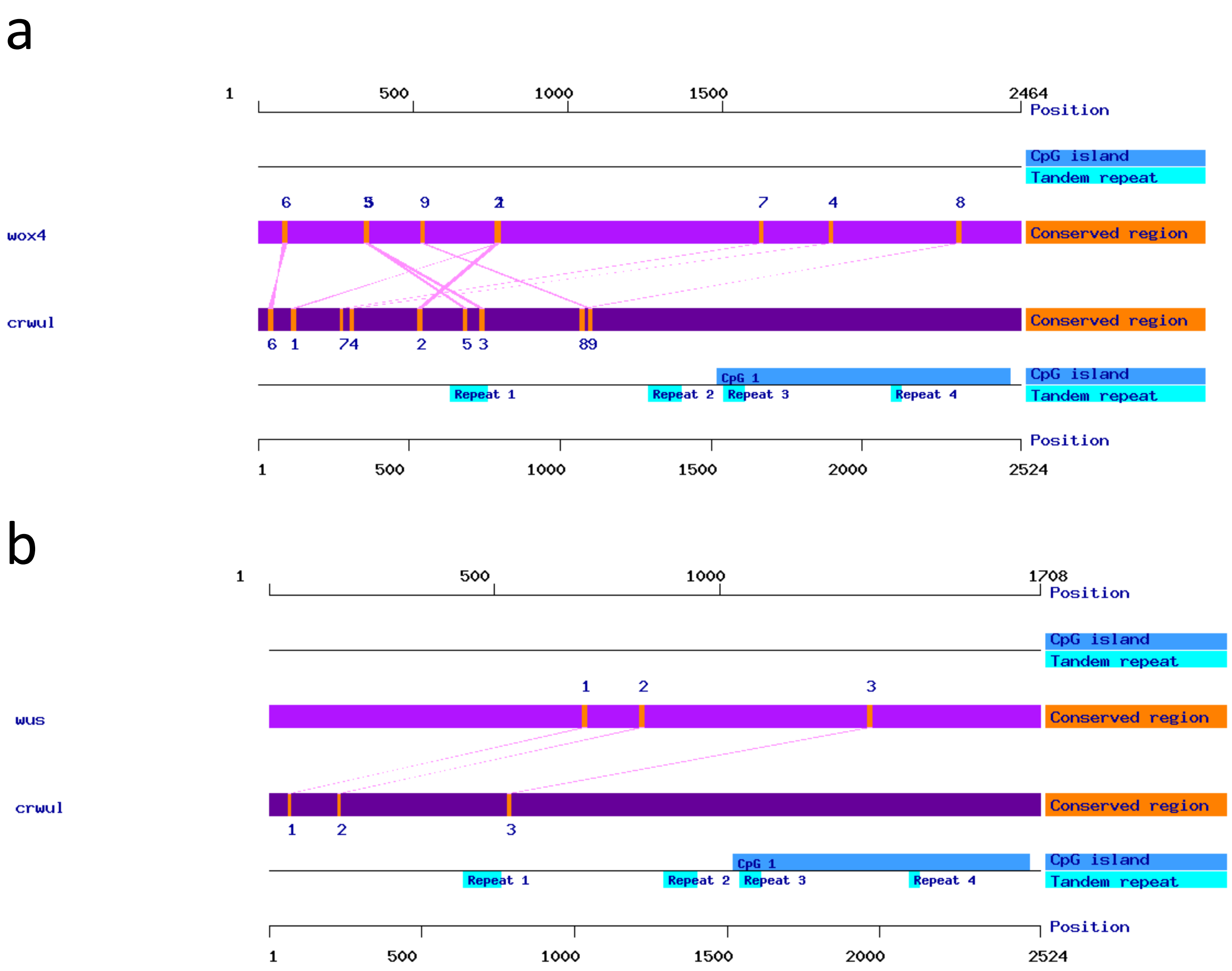

Figure S12: Upstream sequence comparison between *AtWUS*, *CrWUL* and *AtWOX4*. (a) Comparison of *CrWUL* and *AtWOX4* ~2.5kb of upstream sequence. (b) Comparison of *CrWUL* and *AtWUS* ~2.5kb and 1.7kb respectively. Comparisons were made with PlantPAN3.0.
