# Supplementary material for: Vascular function of the T3/modern clade WUSCHEL-Related HOMEOBOX transcription factor genes predate apical meristem-maintenance function
Source: BMC Plant Biol. 2022 Apr 25;22:210. doi: 10.1186/s12870-022-03590-0 (PMC9036803; doi:10.1186/s12870-022-03590-0)
Supplement: Supplementary file 4 — Additional file 4. [file 12870_2022_3590_MOESM4_ESM.pdf]

Supplemental Table S1. Primer sequences used in the study.

Gene	Sequence	Use
CrWUL	F-cacc*cactcgccaatctcaagcat	Insert for RNAi region 1
	R-ctaatccgttctgtgcgataaagg	
	F-cacc*tcagctgaaccgtgatgtag	Insert for RNAi region 2
	R-cttgagattggcgagtgtttg	
	F-atgagagaatatgtgcggggatc	RT-qPCR
	R-attgtagccgtacagagaccac	
	F-cactcgccaatctcaagcat	*In situ* probes anti-sense
	R-taatacgactcactatagg*ctaatccgttctgtgcgataaagg	
	F-taatacgactcactataggg*atgagagaatatgtgcggggatc	*In situ* probes sense
	R-attgtagccgtacagagaccac	
	F-cacc*atgagagaatatgtgcggggatc	Whole mount *in situ* probes anti-sense
	R-taatacgactcactataggg*attgtagccgtacagagaccac	
	F-taatacgactcactataggg*cgaaggagaacatggtaatg	Whole mount *in situ* probes sense
	R-cttgagattggcgagtgtttg	
	F-cacc*atgagagaatatgtgcggggatc	Full-length for *atwox4* rescue
	R-aggaagaacgctaactttcagttc	

Supplemental Table S1. Primer sequences used in the study.

AtWOX4	F-cacc*cgagaaaggcatgcatagcatt	*In situ* probes anti-sense
	R-taatacgactcactataggg*tcatgacttcatctcccttcagga	
	F-taatacgactcactataggg*cgagaaaggcatgcatagcatt	*In situ* probes sense
	R-tcatgacttcatctcccttcagga	
	F-nnnaagctt*ggcaagtgtagtggaggagg	Promoter cloning for *atwox4* rescue
	R-nnnttaattaa*tgctatatgttaaaactagcaaatgctatg	
CrUBQ	F-gatggccgtactcttgcagac	Expression analysis RT-qPCR
	R-ggagacgaagcacgagatga	
CrHistone4	F-taatacgactcactataggg*atgtctggaaggggaaaaggag	*In situ* probes
	R-taatacgactcactataggg*tcagccgccgaagccatagag	
Hygromycin	F-aggtctacccccttttcaacg	Genotyping
	R-aatgtgtgggttcagttggag	
Gabi-Kat	F-aggtctacccccttttcaacg	Genotyping *atwox4* null mutants (Primer Set 1)
	R-aatgtgtgggttcagttggag	
	F-gcaatgagtatgatggtcaatatg	Left border of pAC161 (Primer Set 2)
	R-gggctacactgaattggtagctc	
	F-atattgaccatcatactcattgc	

Supplemental Table S1. Primer sequences used in the study.

	R-aatgtgtgggttcagttggag	o8409 and Set 1 R (primer set 3)
SkWOX11c	F-taatacgactcactatаggg*atggaggcgccaggcac	*In situ* probes
	R-taatacgactcactataggg*tcaggctgcactcccgc	
SkWOX11b	F-taatacgactcactataggg*atgctgccgtgccaagaaagtc	*In situ* probes
	R-taatacgactcactataggg*ttactgctgcagctgctgctg	

Sequences left of * are added for directional cloning or T7 promoter sequence. All sequences are 5' to 3'

Table S2: Statistics for gametophyte cell counts

Dunnett's multiple comparisons test	Predicted (LS) mean diff.	95.00% CI of diff.	Below threshold?	Summary	Adjusted P Value
7					
RN3 vs. crwul1	-22.69	-107.6 to 62.22	No	ns	0.8719
RN3 vs. crwul3	-118.9	-215.7 to -22.06	Yes	*	0.011
RN3 vs. crwul2	-21.22	-118.0 to 75.59	No	ns	0.9233
10					
RN3 vs. crwul1	-96.31	-204.1 to 11.50	No	ns	0.091
RN3 vs. crwul3	-275.4	-376.2 to -174.7	Yes	****	<0.0001
RN3 vs. crwul2	-163.2	-268.6 to -57.77	Yes	***	0.0009
14					
RN3 vs. crwul1	-343.3	-436.1 to -250.4	Yes	****	<0.0001
RN3 vs. crwul3	-266.1	-362.5 to -169.6	Yes	****	<0.0001
RN3 vs. crwul2	-46.01	-134.3 to 42.29	No	ns	0.4749
